# Non-hydrolyzable Diubiquitin Probes Reveal Linkage-Specific Reactivity of Deubiquitylating Enzymes Mediated by S2 Pockets

**DOI:** 10.1016/j.chembiol.2016.03.009

**Published:** 2016-04-21

**Authors:** Dennis Flierman, Gerbrand J. van der Heden van Noort, Reggy Ekkebus, Paul P. Geurink, Tycho E.T. Mevissen, Manuela K. Hospenthal, David Komander, Huib Ovaa

**Affiliations:** 1 Department of Cell Biology II, The Netherlands Cancer Institute, Plesmanlaan 121, 1066 CX Amsterdam, the Netherlands; 2 Medical Research Council Laboratory of Molecular Biology, Cambridge Biomedical Campus, Francis Crick Avenue, Cambridge CB2 0QH, UK

## Abstract

Ubiquitin chains are important post-translational modifications that control a large number of cellular processes. Chains can be formed via different linkages, which determines the type of signal they convey. Deubiquitylating enzymes (DUBs) regulate ubiquitylation status by trimming or removing chains from attached proteins. DUBs can contain several ubiquitin-binding pockets, which confer specificity toward differently linked chains. Most tools for monitoring DUB specificity target binding pockets on opposing sides of the active site; however, some DUBs contain additional pockets. Therefore, reagents targeting additional pockets are essential to fully understand linkage specificity. We report the development of active site-directed probes and fluorogenic substrates, based on non-hydrolyzable diubiquitin, that are equipped with a C-terminal warhead or a fluorogenic activity reporter moiety. We demonstrate that various DUBs in lysates display differential reactivity toward differently linked diubiquitin probes, as exemplified by the proteasome-associated DUB USP14. In addition, OTUD2 and OTUD3 show remarkable linkage-specific reactivity with our diubiquitin-based reagents.

## Highlights

• Protease-resistant diUb probes bind to DUB S1-S2 sites and react at the proximal end
• First kinetic assay showing proximal end cleavage by DUBs using diUb-based substrates
• OTUD3 binds K11-linked diUb and OTUD2 binds K11- and K33-linked diUb in S1-S2 pockets
• Kinetics suggest different mechanisms for polyUb cleavage by OTUD2 and OTUD3

## Graphical Abstract

**Figure:**
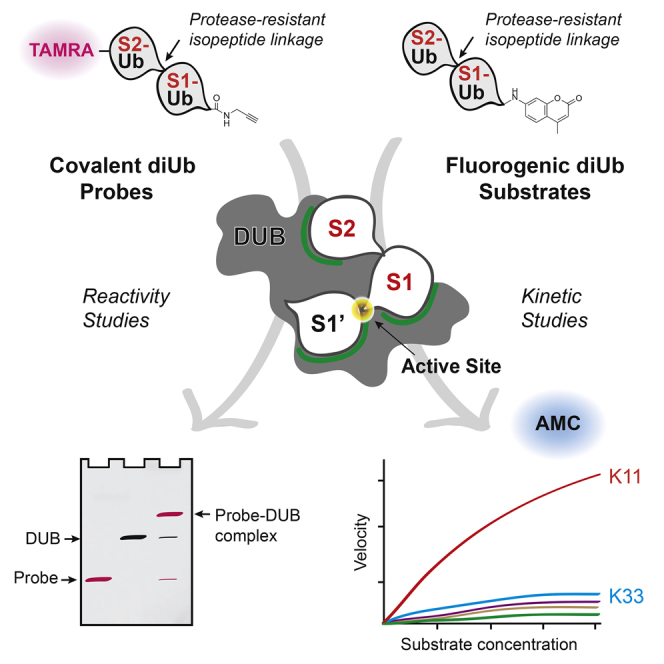

## Teaser

Deubiquitylating enzymes (DUBs) use multiple ubiquitin-binding pockets to disassemble polyubiquitin chains. Most tools probing DUB specificity target pockets adjacent to the active site. The protease-resistant diUb probes described here monitor linkage specificity of an additional S2 pocket and provide kinetics that explain polyubiquitin chain specificity.

## Introduction

Ubiquitin (Ub), a 76 amino acid post-translational modifier, is at the center of a large number of cellular processes. Target proteins can be covalently modified with Ub on either a lysine residue on the protein surface or on the N terminus. First, Ub is activated by an E1 enzyme, forming a thioester bond via its C-terminal carboxylate. Ub is then transferred onto an E2 enzyme, which in conjunction with an E3 enzyme can ubiquitylate a target protein. Ub can also be coupled to another Ub molecule via any of its seven lysine residues or its N terminus to yield Ub chains. Specific combinations of E2 and E3 enzymes dictate substrate specificity and the formation of specifically linked Ub chains (Hershko and Ciechanover, 1998, Hicke et al., 2005, Komander and Rape, 2012, Ye and Rape, 2009). The different linkage types and varying chain lengths determine the transduction of Ub signals through recognition by specific Ub-binding domains in proteins. Deubiquitylating enzymes (DUBs) can reverse ubiquitylation by cleaving the (iso)-peptide bond between the C-terminal carboxylate of Ub and the substrate. Therefore, the ubiquitylation state of a given protein is a delicate balance of ubiquitylation and deubiquitylation events. Approximately 100 human DUBs are known so far, and some DUBs have been shown to exhibit linkage and substrate specificity in the deubiquitylation reaction (Clague et al., 2013, Faesen et al., 2011, Komander et al., 2009, Mevissen et al., 2013).

**Figure 1:**
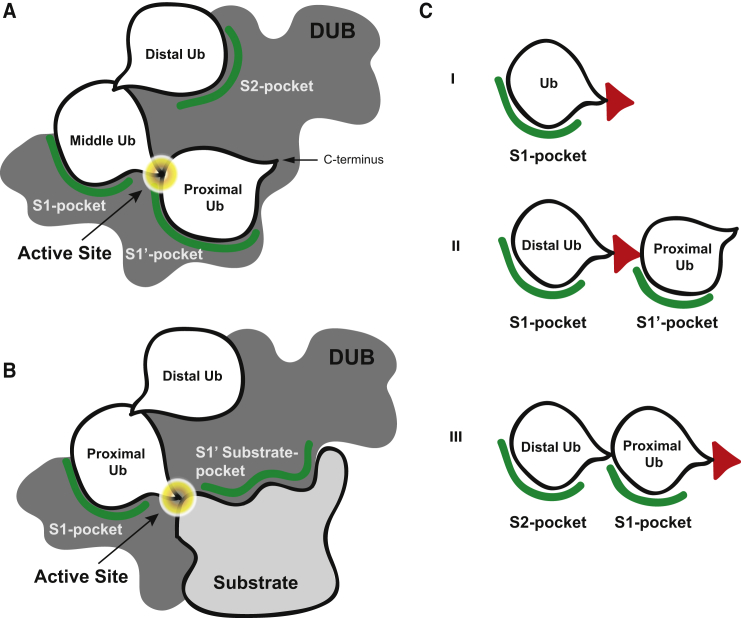
Substrate Specificity Is Controlled by Binding Pockets in DUBs The specificity of DUBs for differently linked Ub chains and ubiquitylated substrates is governed by specific binding pockets. (A) Overview of different pockets that may govern specificity in polyUb chain recognition and processing. (B) Overview of binding pockets that may play a role in recognition and processing of (poly)ubiquitylated substrates. (C) Tools to study the binding and processing of DUBs using the various Ub-binding sites. I, mUb probe/substrate, targeting the S1 pocket; II, isopeptide warhead-containing diUb probes or differently linked diUb substrates, to study S1-S1′ pockets; III, C-terminally reactive diUb-based probes/substrates, targeting S1-S2 pockets, as described in this article. When discussing free Ub chains or diUbs, the most C-terminal Ub in a chain is referred to as proximal and the most N-terminal Ub as distal. The active site reactive element/reporter molecule is depicted as a red triangle.

DUBs can have several modes of action, depending on the type of binding surfaces they contain. Some DUBs can completely disassemble Ub chains, whereas others may be involved in chain editing, in which a chain is partially trimmed before it is modified with a differently linked Ub chain, to form heterotypic chains. These types of DUBs cleave between subsequent Ub modules in a chain and have specific Ub-binding pockets on opposing sides of the active site; one that binds the Ub moiety preceding (S1) and one following (S1′) the scissile bond (Figure 1A). Other DUBs can cleave monoUb (mUb) or Ub chains from protein substrates. These DUBs have an S1 site where the Ub most proximal to the substrate attachment site would bind, but lack an S1′ Ub-binding pocket (Figure 1B). Instead these DUBs may have a specific S1′ substrate-binding pocket. An S2 or even S3 site preceding the S1 Ub-binding pocket may accommodate more distal Ub modules of a chain to enhance specificity further. In addition, it is possible that DUBs that contain an S1′ Ub-binding pocket also contain S2 or even S3 sites. Since DUBs can bind Ub chains utilizing any of these binding pockets, defined tools targeting these different sites are needed to examine chain recognition and cleavage specificity.

Ub-based active site-directed probes have aided the understanding of hydrolytic activity in the Ub system significantly. First-generation probes targeting DUBs were based on a single Ub moiety (Figure 1C-I), relying exclusively on S1 interaction. These probes were instrumental in the identification of many DUBs (Borodovsky et al., 2001, Borodovsky et al., 2002, de Jong et al., 2012, Ekkebus et al., 2013, Hemelaar et al., 2004, Lam et al., 1997, Ovaa, 2007). Second-generation probes based on diUb were developed, which targeted S1-S1′ Ub-binding sites (Iphoefer et al., 2012, Li et al., 2014, McGouran et al., 2013). The probe repertoire has expanded greatly by the introduction of total chemical synthesis of Ub-based reagents (El Oualid et al., 2010). Using this method, more complex diUb-based probes and Ub-based probes bearing substrate context were developed (Haj-Yahya et al., 2014, Kumar et al., 2010, Mulder et al., 2014). For example, diUb probes bearing an electrophilic group between two linked Ub modules (Figure 1C-II) can covalently trap DUBs that bind Ub in an S1-S1′-directed fashion (McGouran et al., 2013, Mulder et al., 2014). Although Ub (chain)-based active site-directed probes and activity reagents have proven excellent tools to both identify and characterize DUB activity and specificity for S1-S1′ cleavage, reagents designed to study S1-S2 site binding and cleavage at the proximal end of a diUb module have been lacking so far. Although it is currently not known whether DUBs can specifically recognize multiple Ub elements to cleave a chain off a substrate at the proximal end, for some human and viral DUBs a specific S2 site has been proposed (Békés et al., 2015, Mevissen et al., 2013, Ratia et al., 2014, Reyes-Turcu et al., 2008, Ye et al., 2011). Previously, a non-hydrolyzable linear diUb-aldehyde probe, based on a bacterially expressed intein construct, was used to demonstrate an S2 site in USP21 (Ye et al., 2011). However, non-hydrolyzable isosteres of isopeptide-linked diUb molecules cannot be expressed directly, and therefore we sought to develop such probes using chemical synthesis. Here, we report the development of such diUb probes with a reactive group at the C terminus (Figure 1C-III), as well as fluorogenic diUb substrates to study the proposed S1-S2 binding sites on DUBs in lysates and on purified recombinant DUBs.

## Results

### DiUb Activity-Based Probe and diUb-AMC Substrate Synthesis

**Figure 2:**
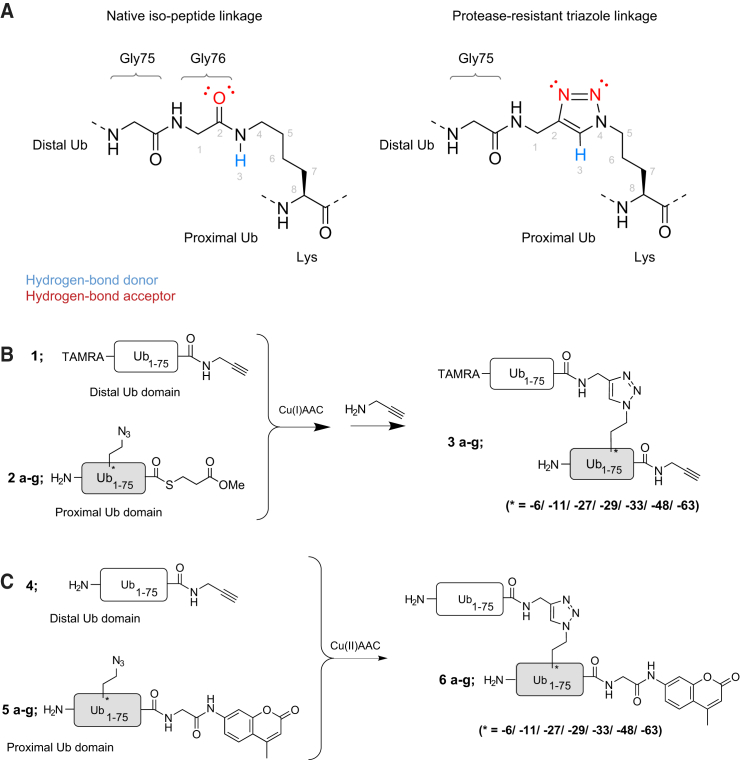
Synthesis of Triazole-Linked Activity-Based Probes (A) Comparison of a native diUb isopeptide linkage and the protease-resistant diUb triazole linkage. (B) Schematic representation of the key CuAAC reaction to generate non-hydrolyzable diUb activity-based probes **3**. (C) Schematic representation of the key CuAAC reaction to generate non-hydrolyzable diUb activity reporter substrates **6**.

**Figure 3:**
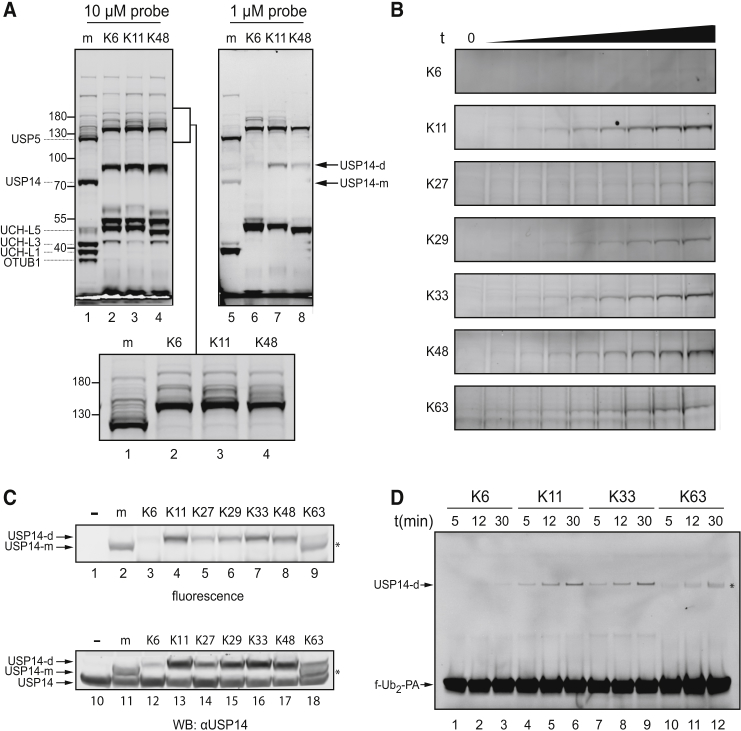
Differential Labeling of DUBs in Lysate with Non-hydrolyzable Probes Targeting S1-S2 Sites (A) EL4 lysate was incubated for 30 min with 10 μM (lanes 1–4) or 1 μM (lanes 5–8) TAMRA-labeled mUb (m), K6-, K11-, or K48-linked diUb probes, and analyzed by SDS-PAGE. The inset shows a magnification of the 120–200 kDa area of the left panel. Approximate molecular weight is indicated in kDa. The identity of the major bands containing DUBs bound to TAMRA-labeled mUb-PA is indicated and was inferred from previous experiments (Borodovsky et al., 2002, Ekkebus et al., 2013). (B) EL4 lysate was incubated with the indicated 5 μM TAMRA-labeled diUb probes. Samples were taken at specific time points over a 30-min time period. The part of the gel representing USP14 is displayed. For full details, see Figure S1A. (C) EL4 lysate was incubated with 3.4 μM TAMRA-labeled mUb-PA or the seven differently linked diUb-PA probes. The top panel shows the fluorescence scan and the bottom panel shows a western blot for USP14 of the same gel. For full gel, see Figure S1B. (D) 0.25 μM TAMRA-labeled K6-, K11-, K33-, and K63-linked diUb probes was incubated for 30 min with 40 nM purified USP14 in the presence of 26S proteasome. Samples were taken at indicated times for analysis. USP14 bound to the TAMRA-labeled mUb (USP14-m) or diUb probe (USP14-d) is indicated. f-Ub_2_-PA indicates the unbound fluorescent diUb probe. *Depending on type of gel and running buffer, some linkages may run at different apparent molecular sizes or display two bands.

To identify and study DUBs with both S1 and S2 Ub-binding pockets, we designed a set of non-hydrolyzable diUb-based active site-directed probes that carry a reactive group (warhead) at the C terminus of the proximal Ub moiety (Figure 1C-III). We decided to use a propargylamide (PA) warhead as it was found to be an excellent warhead to target DUBs (Ekkebus et al., 2013). It provides the broadest reactivity of all Ub-based probes that have been generated so far, and has little reactivity toward enzymes in the Ub conjugation system. Moreover, the stability of the alkyne, commonly used in bio-orthogonal cycloaddition reactions, allows for complex synthetic strategies due to its relative inertness compared with other frequently used warheads. The individual Ub molecules are coupled via a non-hydrolyzable triazole linkage as a peptide bond isostere, preventing unwanted proteolytic cleavage between the two Ub moieties. The triazole linkage, formed by the copper-catalyzed alkyne-azide cycloaddition (CuAAC) reaction between propargylamide and azido-ornithine, is a good isostere of the native glycine-ε-lysine isopeptide bond (Figure 2A). Previously, it was shown that triazole-based polyUb chains and activity-based probes are well tolerated as isopeptide mimics (Dresselhaus et al., 2013, McGouran et al., 2013, Schneider et al., 2014, Weikart et al., 2012). To generate these S1-S2 site-targeting probes, we used a solid-phase peptide-synthesis-based protocol for the linear synthesis of full-length Ub on a chlorotrityl resin (El Oualid et al., 2010). We first cleaved the Ub_1–75_ precursor from the resin using 20% hexafluoroisopropanol (HFIP), which exposed the C-terminal carboxylic acid. The C terminus was then activated and coupled to PA followed by coupling of TAMRA to the N terminus (see Supplemental Experimental Procedures). Subsequently, the Ub molecule was globally deprotected with strong acid and purified by reverse-phase high performance liquid chromatography (RP-HPLC) (Figure 2B; **1**). Using a similar protocol, the proximal Ub reaction partners, equipped with an azido-ornithine mutation at any of the seven lysine positions were synthesized as Ub_1–74_. After liberating the C terminus, the thioester was introduced by coupling of methyl-3-(glycylthio)-propionate. Global deprotection and RP-HPLC purification yielded the thioester precursors (Figure 2B; **2a–g**). The alkyne and azide precursors were coupled in a CuAAC reaction, and subsequently the C-terminal thioesters were converted into the desired PA probe by direct substitution using propargylamine. RP-HPLC purification and size-exclusion chromatography (SEC) were conducted yielding seven TAMRA-diUb-PA probes (Figure 2A; **3a-g**).

**Figure 4:**
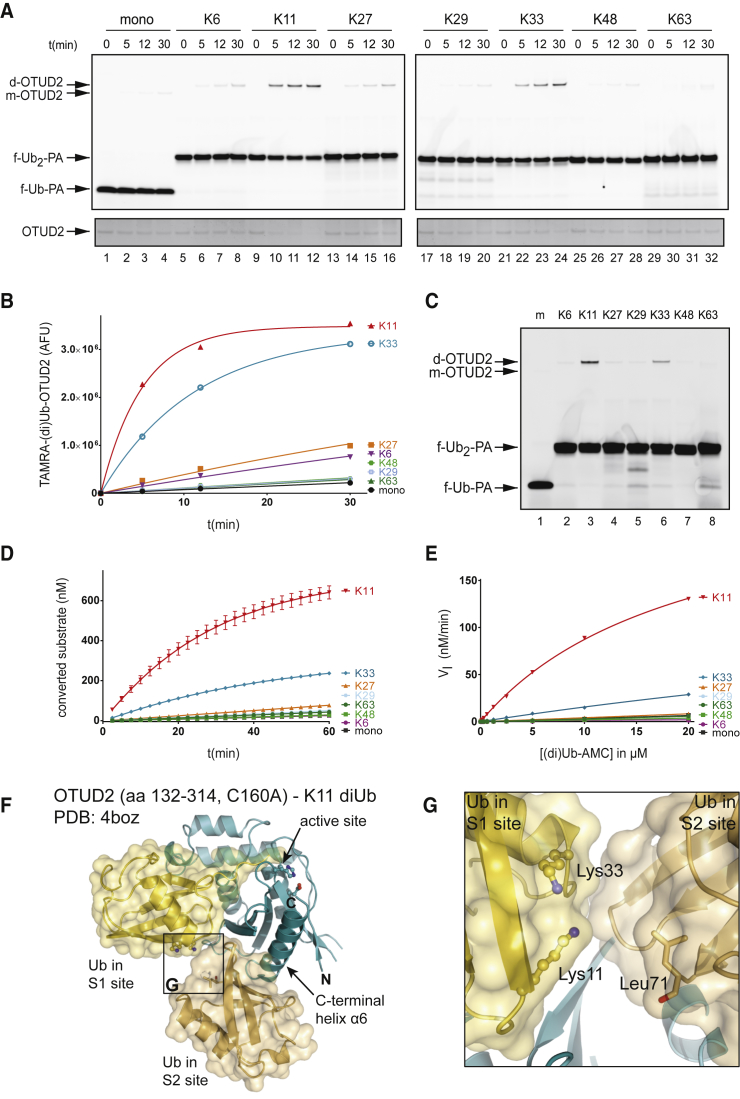
Non-hydrolyzable diUb Probes Reveal the Specificity of OTUD2 for K11 and K33 Ub Linkages (A) 0.2 μM OTUD2 was incubated with 2 μM TAMRA-labeled mUb-PA or diUb-PA probes for indicated times and analyzed by SDS-PAGE. The gel was stained with SYPRO orange (Figure S2A) to visualize unmodified OTUD2 (lower panel). (B) Fluorescent bands containing covalently modified OTUD2 from (A) were quantified, plotted as arbitrary fluorescent units (AFU), and fitted using pseudo-first-order one-phase association kinetics. (C) 0.1 μM OTUD2 was incubated with mUb (m) and diUb probes for 5 min at 1 μM. OTUD2 coupled to TAMRA-labeled mUb-PA (m) or diUb-PA (d) is indicated. f-Ub(_2_)-PA indicates the unbound fluorescent (di)Ub probe. (D) 15 nM OTUD2 was incubated with 2.5 μM (di)Ub-AMC substrate, and fluorescence was measured over time. (E) OTUD2 was incubated with various concentrations of (di)Ub-AMC (Figure S3) as in (D) and initial reaction rates (V_I_) were calculated to generate the Michaelis-Menten plot. (F) Structure of the inactive OTUD2 catalytic domain (aa 132–314, C160A) in complex with K11-linked diUb bound in S1-S2 sites (Mevissen et al., 2013). The Lys11 and Lys33 side chains are not resolved in the electron density maps and were modeled as likely rotamers. (G) Close-up image of the S1-S2 Ub linker region. The last resolved residue of the S2 site Ub C terminus (Leu71) is in close proximity to the Lys11 and Lys33 residues in the Ub moiety bound to the S1 site, suggesting that a K33 linkage might bind similar to the K11 linkage present in this complex. Error bars represent the SD of the mean based on duplicate measurements.

In addition to covalently binding diUb-based PA probes, diUb-fluorogenic substrates were designed to enable further validation of the results obtained with the covalent probes. Ub-7-amido-4-methylcoumarin (Ub-AMC) has been used widely to determine DUB activity (Dang et al., 1998) and allows the determination of kinetic parameters of enzymatic turnover by DUBs. AMC cleavage from Ub liberates fluorescence, and this is therefore a direct measure for DUB activity. DiUb-AMC substrates were generated by equipping the distal Ub molecule with a C-terminal PA moiety through HFIP-mediated local deprotection followed by coupling of propargylamine, global deprotection, and RP-HPLC purification (Figure 2C; **4**). The proximal Ub moiety carrying an azido-ornithine mutation at any of the seven desired positions was prepared as Ub_1–75_ and coupled to glycyl-AMC after HFIP treatment. After strong acid treatment and purification, we obtained the desired series of azide precursors (Figure 2C; **5a–g**). In a CuAAC reaction, the two Ub synthons were coupled and RP-HPLC followed by SEC yielded the complete panel of seven non-hydrolyzable diUb-AMC substrates (Figure 2C; **6a–g**).

### Profiling Enzymes Modified with TAMRA-Labeled diUb Probes in EL4 Cell Lysates

To determine whether the diUb-PA probes can react in a linkage-specific manner, we labeled DUBs present in EL4 mouse lymphoma cell lysates. We showed previously that the mUb-PA probe reacts with a large set of DUBs present in these cells (Ekkebus et al., 2013). In our initial experiment, we compared TAMRA-labeled mUb-PA, and K6-, K11-, and K48- triazole-linked diUb-PA probes to see if differences in specificity could be observed. The K48 linkage has been studied extensively and is involved mainly in proteasomal degradation (Hershko and Ciechanover, 1998). The importance of K11 linkages in mitosis has only recently become clear (Jin et al., 2008), whereas not much is known about the physiological role of K6 linkages. EL4 lysates were incubated with the diUb-PA probes for 30 min at 10 μM and 1 μM (Figure 3A). As expected, bands observed in the diUb-probe-treated lysates (lanes 2–4 and lanes 6–8) run higher than in the mUb-probe-treated lane. At a (di)Ub probe concentration of 10 μM (lanes 1–4), differences between band intensities were minimal, likely because DUBs have already reacted fully. However, some differences were observed in the region containing higher molecular weight DUBs (∼120–200 kDa) as seen in the magnification of this area (inset, lanes 1–4). In lysates treated with 1 μM probe concentration, differences become more pronounced (lanes 5–8), which suggests that distinct DUBs specifically bind and react with differently linked diUb molecules in S1-S2 pockets. Based on the apparent size on gel and western blot analysis, USP14 appeared to be one of the most notable DUBs to display differential reactivity (Figure 3A). Due to different reaction kinetics, differences in specific reactivity for some DUBs may not be fully apparent. Therefore, we repeated this experiment with all seven differently linked diUb probes and took samples at different time points to follow the reactivity of fast-reacting DUBs. However, for some DUBs, kinetic differences cannot be observed as they react too fast under these conditions (Figure S1A). Since USP14 could be easily identified, we show the reaction of USP14 with the different TAMRA-labeled Ub probes in Figure 3B (full details in Figure S1A). USP14 appears to have a preference for reacting with K11, K33, and K48 diUb probes. To verify that USP14 reacted with our probes, we incubated all TAMRA-labeled (di)Ub probes with lysates for 40 min and performed a western blot (Figure 3C, bottom panel). We could show that USP14 indeed reacts with mUb-PA as well as with the diUb-PA probes to variable degrees. Next, we used the TAMRA-labeled diUb-PA probes on purified recombinant USP14 to confirm the results from labeling in lysates. We incubated USP14 in the presence of 26S proteasome, as this is needed for its activation, with K6-, K11-, K33-, and K63-linked diUb-PA probes for the indicated times (Figure 3D). It is clear that the K11-linked diUb probes (lanes 4–6) and the K33-linked diUb probes (lanes 7–9) react much faster than the K6-linked diUb probes (lanes 1–3) and the K63-linked diUb probes (lanes 10–12), similar to the reaction rates observed in lysates. Preferably, we would use our diUb-AMC substrates to do kinetic measurements, but since USP14 is incubated in the presence of 26S proteasome, this obscures the results. The proteasome contains the metallo-DUB RPN11, as well as other Ub-binding proteins, and these interfere with proper kinetic experiments. Therefore, we decided to confirm our S1-S2 diUb probes using other recombinant DUBs in vitro.

### S1-S2 Site-Targeting diUb Probes and Substrates Reveal New Insights into OTUD2 Linkage Specificity

We decided to focus on OTUD2, also known as Yod1, a member of the OTU DUB family, to validate these S1-S2 site-targeting probes. We recently showed that this DUB contains an S2 site in addition to an S1 and S1′ site (Mevissen et al., 2013). We incubated OTUD2 with TAMRA-labeled mUb-PA or diUb-PA probes and followed modification of the enzyme over time. Figure 4A shows that OTUD2 preferentially reacts with the K11-linked diUb probe (lanes 9–12), and to a lesser degree, with the K33-linked diUb probe (lanes 21–24). A higher molecular weight TAMRA-labeled adduct, the expected size of OTUD2 coupled to the diUb-PA probe, is formed over time, whereas the OTUD2 enzyme disappears as expected (lower panel, Figures 4A and S2). Fluorescent bands were quantified and plotted in Figure 4B. The data were fitted using a one-phase association curve. With a t_1/2_ of 3.5 min, the K11-linked diUb probe reacts faster than the K33-linked diUb probe with a t_1/2_ of 8.5 min, and significantly faster than any of the other linkages with a t_1/2_ of 59 min or higher. The experiment was repeated with 0.1 μM OTUD2 and with the different Ub probes at a concentration of 1 μM for 5 min for direct side-by-side comparison (Figure 4C). Clearly, OTUD2 reacts much faster with the K11-linked diUb probe than with the K33-linked diUb probe (lane 3 versus lane 6), whereas the others show no or very limited reactivity.

To further validate the specificity of our reagents targeting S1-S2 sites, we used diUb-AMC reagents **6a–g** (Figure 2B) to establish a kinetic assay to analyze DUB-mediated cleavage at the proximal end of S1-S2 bound diUb substrates. To determine whether specificity could be observed for these substrates, 15 nM OTUD2 was incubated with 2.5 μM mUb-AMC or diUb-AMC, and the increase in fluorescence, due to cleavage of AMC, was measured over time. Figure 4D shows that OTUD2 activity toward K11-linked diUb substrate is higher than toward K33-linked diUb substrate. The other substrates are not processed or processed to a much lesser extent. Next, we incubated OTUD2 with different substrate concentrations and determined initial reaction rates to generate a Michaelis-Menten curve (Figures 4E and S3). The K_M_ for the K11 diUb-AMC substrate was ∼20 μM, whereas the K_M_ for K33 diUb-AMC was ∼100 μM. The K_M_ for mUb-AMC could not be measured and was likely much higher than 100 μM. Apparently, increased affinity plays a role in conferring specificity for the K11 and K33 Ub linkages by OTUD2. Unfortunately, we could not obtain proper V_max_ values for K33-linked diUb-AMC and mUb-AMC since we could only measure the turnover rate at concentrations up to 20 μM. The covalent diUb-PA probes and diUb-AMC substrate experiments confirm the preference of OTUD2 for K11-linked diUb and identify a new preference in OTUD2 for longer K33-linked chains that could not have been detected in diUb-based activity assays previously. These findings can be reconciled from available OTUD2 structures, in particular from a structure of OTUD2 bound to K11-linked diUb, in which Ub moieties interact with S1 and S2 sites of the enzyme (Figure 4F). In this structure, the C terminus of the Ub bound at the S2 site of the enzyme is linked to K11 of the Ub at the S1 site of the enzyme. Importantly, the K11 and K33 residues of the S1-bound Ub are in close proximity (Figure 4G), and it is feasible that a K33-linked diUb molecule would be positioned similarly on OTUD2. This suggests that also K33-linked chains could utilize the OTUD2 S1 and S2 sites for preferential hydrolysis of longer K33-linked chains.

**Figure 5:**
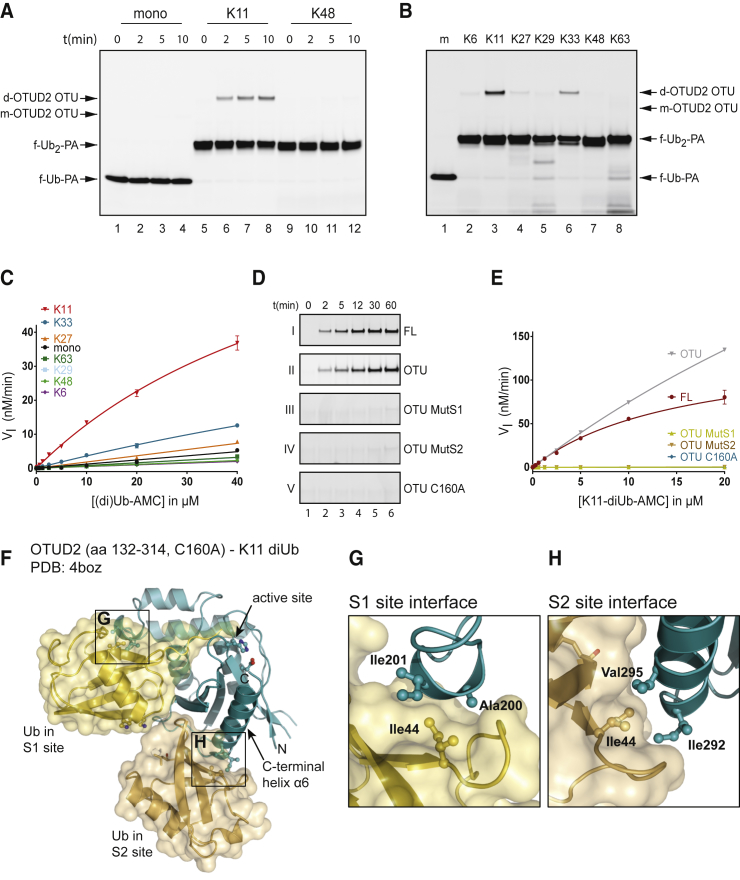
Non-hydrolyzable diUb Probes Specifically Target the S1-S2 Site of OTUD2 (A) The isolated OTU domain of OTUD2 (OTUD OTU) at a concentration of 0.25 μM was incubated with 2.5 μM TAMRA-labeled mUb (m), K11-linked, or K48-linked diUb-PA probes for indicated times. (B) OTUD2 OTU was incubated with TAMRA-labeled mUb-PA or the seven differently linked diUb-PA probes as in (A) for 5 min. OTUD2 OTU coupled to TAMRA-labeled mUb-PA (m) or diUb-PA (d) is indicated. f-Ub(_2_)-PA indicates the unbound fluorescent (di)Ub probe. (C) 15 nM OTUD2 OTU was incubated with Ub-AMC or diUb-AMC substrates at different indicated concentrations, and the increase in fluorescence was measured over time (Figure S4A). The initial reaction rates (V_I_) were calculated to generate the Michaelis-Menten plot. (D) 0.1 μM full-length (FL) OTUD2 or isolated OTU domains of WT OTUD2 (aa 147–314), an S1 site mutant (aa 147–314, AI200-201DD), an S2 site mutant (aa 147–314, I292Q, V295Q), and the catalytically inactive C160A mutant were incubated with 1 μM TAMRA-labeled K11-linked diUb-PA probes. Samples were taken at indicated time points for SDS-PAGE and subsequent fluorescence scanning. All panels were scanned and processed similarly except panel II, which was scanned separately. See Figure S2B for full SYPRO orange stained gels. (E) 15 nM of the OTUD2 variants mentioned in (D) were incubated with various concentrations of K11-linked diUb-AMC substrate. The increase in fluorescence was measured over time (Figure S4B). The initial reaction rates (V_I_) were calculated to generate the Michaelis-Menten plot. (F) Complex structure of OTUD2 and K11-linked diUb as in Figure 4F (Mevissen et al., 2013). (G) Close-up of the S1 site interface. Residues Ala200 and Ile201 were mutated in the OTUD2 OTU MutS1 construct. (H) Close-up image of the S2 site interface. The residues Ile292 and Val295 that interact with the Ub Ile44 patch were substituted in the OTUD2 OTU MutS2 mutant. Error bars represent the SD of the mean based on duplicate measurements.

The observed specificity for K11- and K33-linked diUb probes due to engagement of S1-S2 sites on OTUD2 contrasts with the specificity of OTUD2 in a diUb cleavage assay where Ub binding is governed by the S1 and S1′ pockets. OTUD2 cleaves K11-, K27-, K29-, K33- and to some extent K48-linked diUb, whereas the isolated OTU domain of OTUD2, which lacks the UBX-like and zinc finger domain (ZNF) domain, was shown to have a clear preference for K11-linked diUb (Mevissen et al., 2013). The main determinant for the broader preference of full-length OTUD2 in the diUb cleavage assay was the ZNF domain. To further understand the specificity of the catalytic OTU domain for K11 linkages, we tested the OTUD2 construct lacking both domains to determine if specificity is solely controlled by the S1 and S2 sites within the OTU domain. We refer to this construct, consisting of amino acids 147–314, as OTUD2 OTU. We first incubated OTUD2 OTU with mUb-, K11 diUb-, and K48 diUb-PA probes and followed the modification of the enzyme over time (Figure 5A). We observed that OTUD2 OTU reacts much faster with the K11 diUb probe (lanes 5–8) than with the mUb (lanes 1–4) or the K48 diUb probe (lanes 9–12). Next, we incubated the mUb-PA probe and the full panel of diUb-PA probes with OTUD2 OTU for 5 min. Figure 5B shows that OTUD2 OTU preferentially reacts with the K11 diUb-PA probe (lane 3), although some reactivity with the K33 probe is observed as well (lane 6). The other diUb-PA probes showed no or very limited reactivity. In addition, a (di)Ub-AMC assay was carried out to determine if OTUD2 OTU can preferentially cleave specifically linked diUb-AMC substrates. Here, we see a similar specificity for K11 and K33 diUb linkages as we did with the PA probes (Figures S4A and 5C). Both full-length OTUD2 and OTUD2 OTU display a similar specificity, suggesting that S1-S2 binding is not affected by the ZNF domain or the UBX-like domain. To confirm that our probes indeed targeted the S1 and S2 sites in the OTU domain of OTUD2, we used specific S1 and S2 site mutants (Figures 5F–5H) that were previously shown to diminish processing of a K11-linked trimer Ub chain (Mevissen et al., 2013). In addition, we used the catalytically dead OTUD2 C160A construct to show that probe binding to the enzyme does not occur randomly but requires a functional active site. Different constructs at a concentration of 0.1 μM were incubated with 1 μM TAMRA-labeled K11-linked diUb-PA, and modification of the enzymes was followed over time. Figure 5D shows that both the full-length (FL) OTUD2 and the OTUD2 OTU construct show similar activity with t_1/2_ of 5.3 and 6.8 min, respectively (Figure S2C). In accordance with their modification with the diUb-PA probe, unmodified OTUD2-FL and OTUD2 OTU disappear on the gel (Figure S2B). In contrast, the S1 and the S2 mutant OTUD2 OTU constructs showed very little reactivity, and only after 60 min was a minor band observed. In addition, the C160A construct did not show any labeling at all. Similar results were obtained using the K11-linked diUb-AMC substrate. The OTUD2-FL and the OTUD2 OTU constructs process the substrate much more efficiently than the S1 and S2 mutant OTUD2 OTU constructs, whereas the C160A construct does not show any activity at all (Figure 5E). These results clearly show that our diUb reagents target the S1 and S2 sites on OTUD2. Likely, OTUD2 utilizes all available Ub-binding pockets (i.e., S2, S1, and S1′) to increase specificity for K11- and K33-linked polyUb chains.

### An S2 Ub-Binding Site in the OTU Domain of OTUD3 Confers Specificity for K11 Linkages

**Figure 6:**
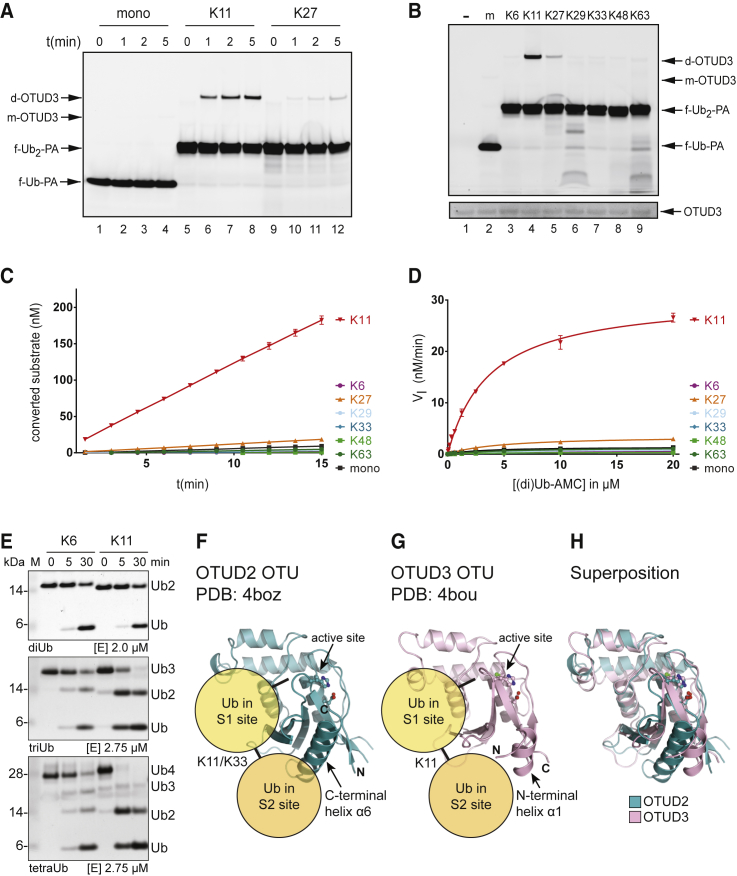
OTUD3 Displays K11 Linkage Specificity Using S1-S2 Site Probes and Substrates (A) 0.15 μM OTUD3 was incubated with 1 μM TAMRA-labeled mUb-PA, K11-linked diUb-PA, or K27-linked diUb-PA probes for indicated times, and analyzed by SDS-PAGE and fluorescence scanning. (B) OTUD3 was incubated with buffer (−), TAMRA-labeled mUb-PA (m), or the seven differently linked diUb-PA probes for 4 min at similar concentrations as in (A). Unmodified OTUD3 was visualized by SYPRO orange staining (Figure S5). OTUD3 coupled to mUb-PA (m) or diUb-PA (d) is indicated. f-Ub(_2_)-PA indicates the unbound fluorescent (di)Ub probe. (C) 15 nM OTUD3 was incubated with the different (di)Ub-AMC substrates at a concentration of 2.5 μM, and activity was measured over time. (D) OTUD3 OTU was incubated with various concentrations of (di)Ub-AMC (Figure S6A) as in (C) and initial reaction rates (V_I_) were calculated to generate the Michaelis-Menten plot. (E) In vitro polyUb cleavage assay. OTUD3 OTU domain (aa 52–209) was incubated with K6- and K11-linked diUb (top panel), triUb (middle panel), and tetraUb (bottom panel). Assays were performed at indicated enzyme concentrations. Samples were taken at indicated times for analysis by SDS-PAGE and silver staining. M, marker. (F–H) Structures of the (F) OTUD2 OTU domain from the K11 diUb complex shown in Figure 4F, (G) the OTUD3 OTU domain (Mevissen et al., 2013), and (H) superposition of both structures. The position of a diUb molecule bound in S1-S2 sites is shown in (F) and (G). The C-terminal helix in OTUD2 and the structurally equivalent N-terminal helix in OTUD3 are indicated. Error bars represent the SD of the mean based on duplicate measurements.

OTUD3, another DUB of the OTU family that has remained uncharacterized, was shown to contain an OTU domain that preferentially hydrolyzed K6- and K11-linked diUb. The distinct cleavage profiles of the isolated OTU domains of OTUD2 and OTUD3 was striking, in particular since both were structurally similar (Mevissen et al., 2013). However, the diUb cleavage assay only determines specificity imposed by the S1-S1′ Ub-binding pockets and cannot inform on potential S2 Ub-binding sites. Whether OTUD3, like OTUD2, also prefers longer substrates that occupy the S1 and a putative S2 site is unknown. Therefore, we incubated OTUD3 with the different TAMRA-labeled (di)Ub-PA probes. In our initial experiment, we compared reaction kinetics of mUb-PA, K11-, and K27-linked diUb-PA probes. Figure 6A shows that OTUD3 preferentially reacts with the K11-linked diUb probe (lanes 5–8), and that the reaction with the K27-linked diUb probe proceeds much slower (lanes 9–12). To further characterize the linkage specificity, the experiment was repeated with all (di)Ub probes under similar conditions to compare all linkages side by side. Figure 6B shows that OTUD3 preferentially reacts with the K11-linked diUb-PA probe (lane 4), although some reactivity of OTUD3 with the K27-linked diUb probe is observed as well (lane 5). The other diUb-PA probes did not show any or very limited reactivity. Interestingly, this also applies to the K6-linked diUb-PA probe, despite the preference of the enzyme for K6-linked diUb in cleavage assays.

Experiments were carried out with (di)Ub-AMC substrates to further examine the specificity of OTUD3 for the S1-S2 site probes. OTUD3 (15 nM) was incubated with 2.5 μM substrates. We observe that K11-linked diUb-AMC is preferentially cleaved over all other linkages (Figure 6C). Interestingly, in this experiment, K27-linked diUb-AMC does not appear to be cleaved very effectively compared with Figures 6A and 6B. Possibly, this is due to the different substrate concentrations used in these experiments. To determine kinetic parameters for the reaction, we incubated OTUD3 with different substrate concentrations and determined the initial reaction rates, from which a Michaelis-Menten curve could be generated (Figures 6D and S6A). From these experiments, it is evident that K11-linked diUb is the preferred substrate for OTUD3. The K_M_ for K11-linked diUb-AMC is 3.7 μM, which is similar to the K_M_ of mUb-AMC (3.1 μM) and K27-linked diUb-AMC (4.7 μM). Interestingly, the observed K11 specificity is due to a difference in V_max_ (Figures 6D and S6B). The V_max_ for K11-linked diUb-AMC is 30.8 nM/min, whereas the V_max_ for K27-linked diUb-AMC and the mUb-AMC are 3.6 and 1.4 nM/min, respectively. This also explains the differences observed between Figures 6A and 6C, where 1 μM versus 2.5 μM substrate was used, respectively. Also in the diUb-AMC assay, the K6 linkage was not hydrolyzed by OTUD3 (Figures 6C and 6D). Together, this suggests that the linkage preference of OTUD3 is multi-layered; the S1-S1′ sites impose a preference for K6 and K11 linkages, and the additional S2 site sharpens this preference to target longer K11-linked Ub chains. If the S2 site on OTUD3 contributes to increased K11 specificity, this should be visible when longer polyUb substrates are used. Indeed, when OTUD3 was incubated with K6- and K11-linked di-, tri-, and tetraUb, clear differences in chain hydrolysis were observed for the cleavage of longer (n > 2) Ub chains (Figure 6E). While longer K6-linked chains were hydrolyzed independently of their length, K11-linked tri- and tetraUb was hydrolyzed significantly faster. The slower kinetics for K11-linked diUb likely arises from loss of the S2 site contribution but could also be due to alternating binding of K11 diUb to sites on OTUD3, where the diUb can bind either productively to S1-S1′ or non-productively to S1-S2 sites. A direct comparison between S1-S2-mediated diUb-AMC hydrolysis and the S1-S1′-mediated diUb cleavage shows that the S1-S2-mediated reaction proceeds at least 30-fold faster (Figure S5), which could perhaps explain the slow diUb hydrolysis. However, for the structurally similar OTUD2, non-productive binding to S1-S2 does not significantly inhibit diUb hydrolysis in S1-S1′ as the wild-type OTUD2 and the S2 site mutant of OTUD2 show similar diUb cleavage kinetics (Mevissen et al., 2013). Although OTUD2 and OTUD3 are structurally similar in their OTU domain (Figures 6F–6H), they do display differences in specificity, especially for K33-linked diUb, and in their mechanism of activation. For OTUD2, the main determinant for S1-S2 site specificity appears to be affinity, whereas for OTUD3 specificity appears to be driven by an increase in V_max_ of the reaction.

## Discussion

Different combinations of E2 and E3 enzymes have been shown to generate specifically linked Ub chains, as either homotypic (linked via the same lysine residue of each Ub) or heterotypic chains (in which single chains contain multiple linkage types forming mixed and branched structures). It is likely, given the complexity of chains and the large amount of known DUBs (∼100), that specificity for disassembly of chains is also common. For some DUBs, specificity toward certain linkage types has been shown (Békés et al., 2015, Mevissen et al., 2013, Ratia et al., 2014, Ritorto et al., 2014), while other DUBs lacked specificity (Faesen et al., 2011). However, such specificity was mostly assayed by monitoring cleavage of diUb, which only reports on Ub-binding sites on either side of the active site of the DUB, i.e., S1-S1′ sites (Figure 1). Importantly, many DUBs contain additional Ub-binding domains, which could potentially act as S2, S3 sites, or alternatively as S2′, S3′ sites. Experimentally verified S2/S3 sites exist in USP5 (Reyes-Turcu et al., 2008) and OTUD2 as we have shown previously (Mevissen et al., 2013). Here, we use new tools to experimentally reveal S2 sites in DUBs and show how these S2 sites sharpen substrate preference in OTU enzymes. Our diUb-PA probes, designed to bind DUBs in the S1 and S2 pockets, show a clear specificity of OTUD2 for K11-linked diUb-PA probes (Figure 4) and to a lesser extent for K33. Previously, this K33 selectivity could not be demonstrated as K33-linked polyUb chains were not available until recently (Kristariyanto et al., 2015, Michel et al., 2015). The diUb-AMC substrates we developed also show similar selectivity for K11- and K33-linked diUb, and enable direct quantitative measurements of the impact of S2-mediated chain hydrolysis. Furthermore, we show that our probes indeed bind the S1 and S2 sites on OTUD2, since mutations in these sites diminished activity toward the K11-linked diUb probes (Figure 5). Hence, the addition of an S2 Ub-binding pocket refines the specificity profile of OTUD2 to target longer K11- and K33-linked Ub chains. Previously, we showed that OTUD2 processes K11-linked polymers much faster than K11-linked diUb, and that this depends on a functional S2 Ub-binding site (Mevissen et al., 2013).

OTUD2 contains a K11-specific OTU domain, and the broader preference (K11, K27, K29, K33) of the full-length enzyme in the diUb cleavage assay appeared to be due to the presence of a C-terminal zinc finger (ZNF) domain (Mevissen et al., 2013). Here, we show that both full-length OTUD2 and the isolated OTU domain of OTUD2 display similar specificity (Figures 4 and 5), suggesting that neither the ZNF domain nor the UBX-like domain in OTUD2 interfere with S1-S2 site specificity. Likely, the S1-S2 sites are a main determinant for conferring linkage specificity. The role of the ZNF domain remains unclear; we could not detect an interaction with Ub in nuclear magnetic resonance analysis (Mevissen et al., 2013), but it is possible that it binds protein substrates. The UBX-like domain of OTUD2 binds AAA+ ATPase p97, an important Ub-dependent regulator of protein homeostasis (Ernst et al., 2009). The deletion of the ZNF domain in catalytically inactive OTUD2 C160S rescued the degradation of an ER-associated degradation substrate (Ernst et al., 2009), which suggests that the ZNF domain is indeed recruiting proteins to p97. In in vitro diUb cleavage experiments, where such putative binding partners are not present, the ZNF domain may therefore compromise specificity when probing the S1-S1′ site. Our previous data combined with the results presented here suggest that OTUD2 is specific for K11-linked chains, and to a lesser extent for K33, and that OTUD2 utilizes S2, S1, and S1′ Ub-binding pockets to govern specificity. Intriguingly, we have just described an HECT E3 ligase, AREL1, which generates K11- and K33-linked polyUb preferentially (Michel et al., 2015). It will be interesting to see if there is a mechanistic or functional connection between these findings.

For OTUD3, we uncover a new aspect of specificity for K11 linkages involving a previously unnoticed S2 site. Previously, OTUD3 was shown to preferentially cleave K6- and K11-linked diUb (Mevissen et al., 2013). We did not observe any reactivity toward K6 diUb; however, the K11 specificity previously found for OTUD3 could be confirmed with our S1-S2 site probes. This suggest that OTUD3 utilizes S2-S1-S1′ binding to confer specificity for polyUb chains linked through K11. Interestingly, our S1-S2 site probes do not show reactivity with the K6 linkage, although a K6-linked diUb that binds in S1-S1′ can be cleaved. An intriguing possibility is that OTUD3 combines the S1-S2 site specificity for the K11 linkage with the S1-S1′ site specificity for the K6 linkage to recognize and process heterotypically linked K6-K11 chains. Whether this is indeed the case needs to be examined further, yet it does stress the importance of using different types of probes that target S1-S1′ and S1-S2 sites to study DUB specificity for polyUb chains.

Structures of OTUD2 are consistent with a K11-specific S2 binding site, and reveal why a K33-linked probe could interact with OTUD2 (Figures 4F and 4G). Interestingly, an analogous binding site could exist in OTUD3, where a similarly placed helix could form the S2 site (Figures 6F–6H). This could lead to a similar recognition of K11-linked diUb in both enzymes, explaining their K11-specific S2 site. However, only OTUD2 cross-reacts with K33-diUb probes, suggesting that the Ub-binding mode in OTUD3 is different enough that this cross-reactivity is no longer present. A further interesting finding relates to the kinetic parameters obtained with diUb-AMC reagents, which show an increase in V_max_, rather than K_M_, and suggest that diUb binding may reorganize and optimize the catalytic center of OTUD3. Future structural studies, for which the diUb probes reported here will be instrumental, may reveal the molecular basis for this curious kinetic behavior.

Another reason these probes are important in our understanding of Ub chain binding to DUBs, is that DUBs containing S2, S1, and S1′ sites could potentially bind diUb modules in any of these sites. When looking at a single type of probe, either targeting S1-S2 or S1-S1′, this may obscure the results; for example, non-productive binding of a diUb to S1-S2 may inhibit the cleavage of diUb in the S1-S1′ site. For OTUD2 and OTUD3, it is likely that the S1-S2 sites are the main determinants for polyUb binding and cleavage. We observed that reaction rates for S1-S2-mediated diUb-AMC hydrolysis by OTUD2 (Figures 4D and 4E) are much higher than the rate of diUb cleavage (Mevissen et al., 2013). For OTUD3, we directly compared rates of S1-S2-mediated diUb-AMC hydrolysis and S1-S1′-mediated diUb cleavage, which showed a difference in half-time of at least 30-fold between diUb-AMC versus TAMRA-labeled diUb hydrolysis (Figure S5). In these cases, it is unlikely that the transient binding in S1-S1′ causes a significant inhibition of S1-S2 binding. On the other hand, the OTUD2 S2 mutant did not show a significant change in diUb cleavage rates, which suggests that non-productive binding to S1-S2 also does not significantly inhibit S1-S1′ hydrolysis. Perhaps the general slow hydrolysis of diUb is an intrinsic property of DUBs targeting polyUb chains. For DUBs containing multiple Ub-binding sites, it is necessary to use different types of probes to study these sites, especially if the sites display large differences in affinity. We conclude that our probes are reagents suitable for targeting the S1-S2 sites of DUBs and will become valuable reagents to study the different Ub-binding sites in DUBs. In addition, the covalent probes may also be a great tool in structural studies for understanding the mechanistics of polyUb recognition and processing. It is conceivable that DUBs that have so far been described as non-specific in assays mainly targeting S1-S1′ sites may in fact display specificity with these S1-S2 site probes. Specificity for polyUb is likely conferred by the binding of multiple Ub moieties in a chain to multiple Ub-binding pockets on a DUB.

The identification of additional Ub-binding sites on DUBs is of physiological relevance, as it suggests that different targeting mechanisms are in operation. DUBs can target ubiquitylated proteins to remove mUb or polyUb chains from them, or may target Ub chains for trimming or complete disassembly. DUBs containing additional Ub-binding sites, such as S2 sites, could act as de-branching enzymes to simplify randomly generated heterotypic polyUb chains. Such activity may exist for example in OTUD3, which may bind via its OTU domain to K6 linkages that are extended by a K11 linkage on the distal Ub. Currently, we lack much understanding of these undoubtedly highly important intricacies of the Ub system. To address these issues, a combination of different Ub-based probes is essential to determine DUB linkage specificity, differentiate DUB action on specific targets, and to fully understand the contribution of DUBs in remodeling the ubiquitome. The probes we have developed here will serve as a crucial tool to contribute to breaking the Ub code.

## Significance

**Determining Ub linkage specificity of DUBs has previously been done mainly using assays that only target the S1 and S1′ pockets adjacent to the active site. This hydrolysis is generally slow, and it has been shown that some DUBs cleave polyUb chains at accelerated rates, suggesting additional Ub-binding pockets. For few DUBs, an S2 pocket preceding the S1 pocket is known. It is likely that more DUBs contain such an S2 pocket but tools to assay this were not available thus far. Our covalent probes or AMC substrates, based on protease-resistant diUb modules, can specifically target S1-S2 pockets on DUBs. Kinetic experiments can be done using the substrates and will be instrumental in elucidating the activation mechanism of DUBs in processing polyUb chains. In addition, covalent probes can be used in structural studies to corroborate these findings. We already showed that specificities for S1-S2 pockets may differ from the S1-S1′ pockets, and thus these tools will be instrumental in evaluating specificity for homotypic or heterotypic polyUb chains.**

## Experimental Procedures

### TAMRA-Labeled Probe In Vitro Assays

For the assays with recombinant purified proteins, the enzymes were diluted at 2× final concentration in 20 mM Tris-HCl (pH 7.5), 150 mM NaCl, 0.1 mg/ml BSA, and 2 mM DTT. The (di)Ub-TAMRA propargylamide probes were diluted at 2× final concentration in 20 mM Tris-HCl (pH 7.5), 150 mM NaCl. For the time course, equal volumes of enzyme and probe were mixed and incubated while shaking at 30°C. At the indicated time points, 10 μl was taken and added to a tube with 5 μl of 4× Laemmli sample buffer (SB). For t = 0, 5 μl of the 2× mix of enzyme or probe was added to 5 μl of 4× SB directly. For the experiment, in which all linkages were incubated at one time point, 5 μl of 2× probe and 2× enzyme were mixed, and the reaction was stopped by the addition of 5 μl of 4× SB. Samples were heated to 65°C, loaded onto a 4%–12% NuPage Bis-Tris gel, and run in 2-(N-morpholino)ethanesulfonic acid (MES) buffer. Final enzyme concentrations are indicated in the figure legends.

For the time course experiments in EL4 lysates (Figures S2A and 3), 90 μl of lysate (60 × 10^6^ cells/ml) was incubated with 90 μl of TAMRA-labeled (di)Ub-PA probe (∼10 μM). At each time point, 20 μl was taken and mixed with 10 μl of 4× SB. The samples were heated to 65°C, loaded on a 10-well 8% Bold gel and run in 3-(N-morpholino)propanesulfonic acid buffer. Gel analysis was done on the ProXpress Imaging system (PerkinElmer) at Em/Ex of 550/590 nm. For Figures 4A, 4B, 5D, and S2C, bands were quantified using Image Studio Lite (Licor Biosciences). Data were fitted using GraphPad Prism 6, and curves were forced to have the same plateau as the highest value found (the K11 linkage), which represented maximal enzyme labeling.

### Fluorogenic Substrate Conversion Assay

A stock solution of diUb-AMC substrate (K63-, K48-, K33-, K29-, K27-, K11-, K6-linked) and monoUb-AMC as reference was diluted to 2× final concentration in assay buffer (50 mM Tris-HCl [pH 7.5], 100 mM NaCl [pH 7.6], 1 mg/ml 3-[(3-cholamidopropyl)-dimethylammonio]-1-propanesulfonate, 2 mM DTT, and 0.5 mg/ml bovine γ-globulin). 10 μl of the respective substrates was added to 10 μl of the 2× final concentration of DUB in assay buffer, incubated in a 384-well assay plate (low volume, flat bottom, non-binding surface, black polystyrene, 3,820; Corning) and analyzed over time using a CLARIOstar (BMG Labtech) spectrophotometer. The final DUB concentration was 15 nM. Fluorescent intensity was measured over time at Ex 360/20, Em 450/30. Duplicates were measured and analyzed using GraphPad Prism software. Error bars represent the SD. The initial reaction rates were calculated from the first 15 min (when the reaction is still linear) and were plotted against diUb-AMC substrate concentration to obtain the Michaelis-Menten plot. Fluorescence intensity values were correlated to concentration of converted substrate by comparing with a standard curve serial dilution of AMC/Ub-AMC (20/0 μM to 0/20 μM) in assay buffer.

### Cell Lysate Preparation

EL4 mouse lymphoma cells were grown in DMEM supplemented with 10% fetal calf serum. Cells were grown to ∼1 × 10^6^ cells/ml and lysates were prepared by taking the cells up in 20 mM Tris-HCl (pH 7.5), 150 mM NaCl, and 0.5% Triton X-100 at 60 × 10^6^ cells/ml. Cells were sonicated for 10 min using a 30-s off/on cycle. After sonication, samples were centrifuged at 21,000 × *g* for 30 min, and the supernatant was used as the resulting cell lysate. DTT was added to a final concentration of 2 mM.

### In Vitro DUB Cleavage Assay

Qualitative in vitro DUB specificity assays of OTUD3 (Figure 6E) with K6- and K11-linked di-, tri-, and tetraUb were performed as described previously (Hospenthal et al., 2015). In short, 2× concentrated ubiquitin stocks (∼10 μM di-,tri-, or tetraUb) were mixed with 2× concentrated stocks of OTUD3 and incubated at 37°C. At indicated time points, samples were taken for SDS-PAGE and silver staining. Samples were run on a 4%–12% gradient gel in MES buffer. Final enzyme concentrations are indicated in the figure.

### Synthesis of Probes

The synthesis of ubiquitin-based probes is detailed in the Supplemental Information. In short, Ub or Ub mutants were generated through linear peptide synthesis. DiUb-based probes were made using a CuAAC-reaction between PA and azido-ornithine yielding a protease-resistant triazole linkage.

### Recombinant DUBs

Human USP14 (+26S proteasome) was purchased from Ubiquigent. Full-length human OTUD2, OTUD2 OTU (aa 147–314), the S1 site mutant (aa 147–314, AI200-201DD), the S2 site mutant (aa 147–314, I292Q, V295Q), the C160A constructs, and OTUD3 (aa 52–209) were described previously (Hospenthal et al., 2013, Mevissen et al., 2013).

### Miscellaneous Materials

The USP14 antibody was purchased from Cell Signaling Technology.

## Author Contributions

R.E. and G.J.v.d.H.v.N. synthesized the probes. P.P.G. designed the reagents, D.F. and G.J.v.d.H.v.N. validated the enzyme specificity. T.E.T.M. and D.K. provided enzymes for this study. M.K.H. performed the OTUD3 polyUb cleavage assay. H.O. guided the overall experimental design. G.J.v.d.H.v.N. and D.F. wrote the manuscript. All authors have read and commented on the manuscript. R.E., D.F., G.J.v.d.H.v.N., P.P.G., T.E.T.M., and M.K.H. declare no competing financial interests. H.O. is founder and shareholder of the company UbiQ that markets reagents in the Ub field. D.K. and H.O. are part of the DUB Alliance that includes Cancer Research Technology and FORMA Therapeutics.

## Supplemental Information

Document S1. Supplemental Experimental Procedures, Figures S1–S6, and Schemes S1 and S2

Data S1. Analytical Data of diUb-PA Probes and diUb-AMC Substrates

Document S2. Article plus Supplemental Information
