# Supplementary material for: Non-hydrolyzable Diubiquitin Probes Reveal Linkage-Specific Reactivity of Deubiquitylating Enzymes Mediated by S2 Pockets
Source: Cell Chem Biol. 2016 Apr 21;23(4):472–82. doi: 10.1016/j.chembiol.2016.03.009 (PMC4850247; doi:10.1016/j.chembiol.2016.03.009)
Supplement: Document S1. Supplemental Experimental Procedures, Figures S1–S6, and Schemes S1 and S2 [file mmc1.pdf]

Cell Chemical Biology, Volume *23*

Supplemental Information

Non-hydrolyzable Diubiquitin Probes Reveal Linkage-Specific Reactivity of Deubiquitylating Enzymes Mediated by S2 Pockets

Dennis Flierman, Gerbrand J. van der Heden van Noort, Reggy Ekkebus, Paul P. Geurink, Tycho E. T. Mevissen, Manuela K. Hospenthal, David Komander, and Huib Ovaa

Figure S1

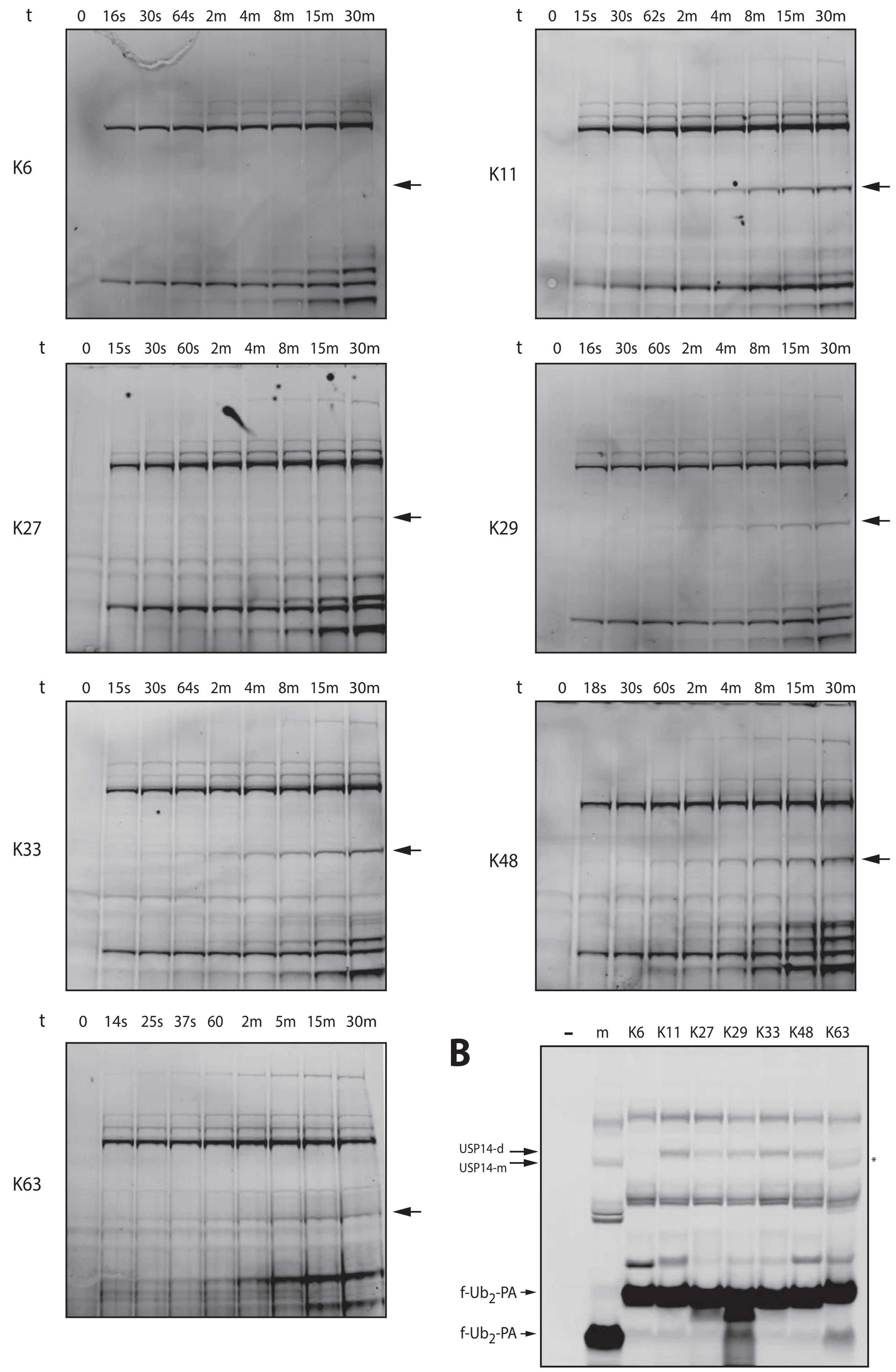
A
t 0 16s 30s 64s 2m 4m 8m 15m 30m
K6
t 0 15s 30s 62s 2m 4m 8m 15m 30m
K11
t 0 15s 30s 60s 2m 4m 8m 15m 30m
K27
t 0 16s 30s 60s 2m 4m 8m 15m 30m
K29
t 0 15s 30s 64s 2m 4m 8m 15m 30m
K33
t 0 18s 30s 60s 2m 4m 8m 15m 30m
K48
t 0 14s 25s 37s 60 2m 5m 15m 30m
K63
B
- m K6 K11 K27 K29 K33 K48 K63
USP14-d
USP14-m
*
f-Ub2-PA
f-Ub2-PA

Figure S2

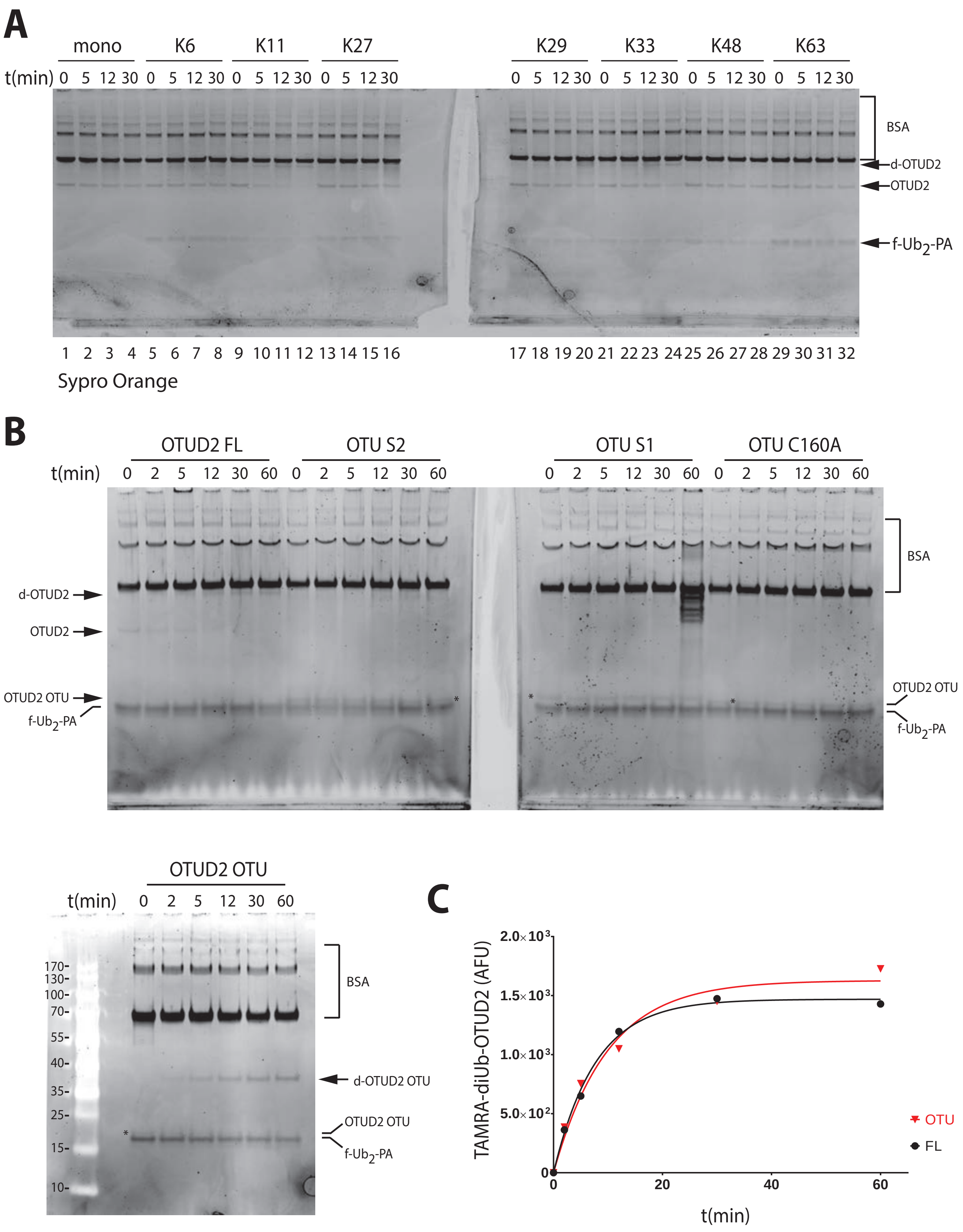
A
mono
K6
K11
K27
K29
K33
K48
K63
t(min) 0 5 12 30 0 5 12 30 0 5 12 30 0 5 12 30
0 5 12 30 0 5 12 30 0 5 12 30 0 5 12 30
BSA
d-OTUD2
OTUD2
f-Ub2-PA
1 2 3 4 5 6 7 8 9 10 11 12 13 14 15 16
17 18 19 20 21 22 23 24 25 26 27 28 29 30 31 32
Sypro Orange
B
OTUD2 FL
OTU S2
OTU S1
OTU C160A
t(min) 0 2 5 12 30 60 0 2 5 12 30 60
0 2 5 12 30 60 0 2 5 12 30 60
d-OTUD2
OTUD2
OTUD2 OTU
f-Ub2-PA
BSA
OTUD2 OTU
f-Ub2-PA
OTUD2 OTU
t(min) 0 2 5 12 30 60
170-
130-
100-
70-
55-
40-
35-
25-
15-
10-
BSA
d-OTUD2 OTU
OTUD2 OTU
f-Ub2-PA
C
TAMRA-diUb-OTUD2 (AFU)
2.0×10³
1.5×10³
1.0×10³
5.0×10²
0
0
20
40
60
t(min)
OTU
FL

Figure S3

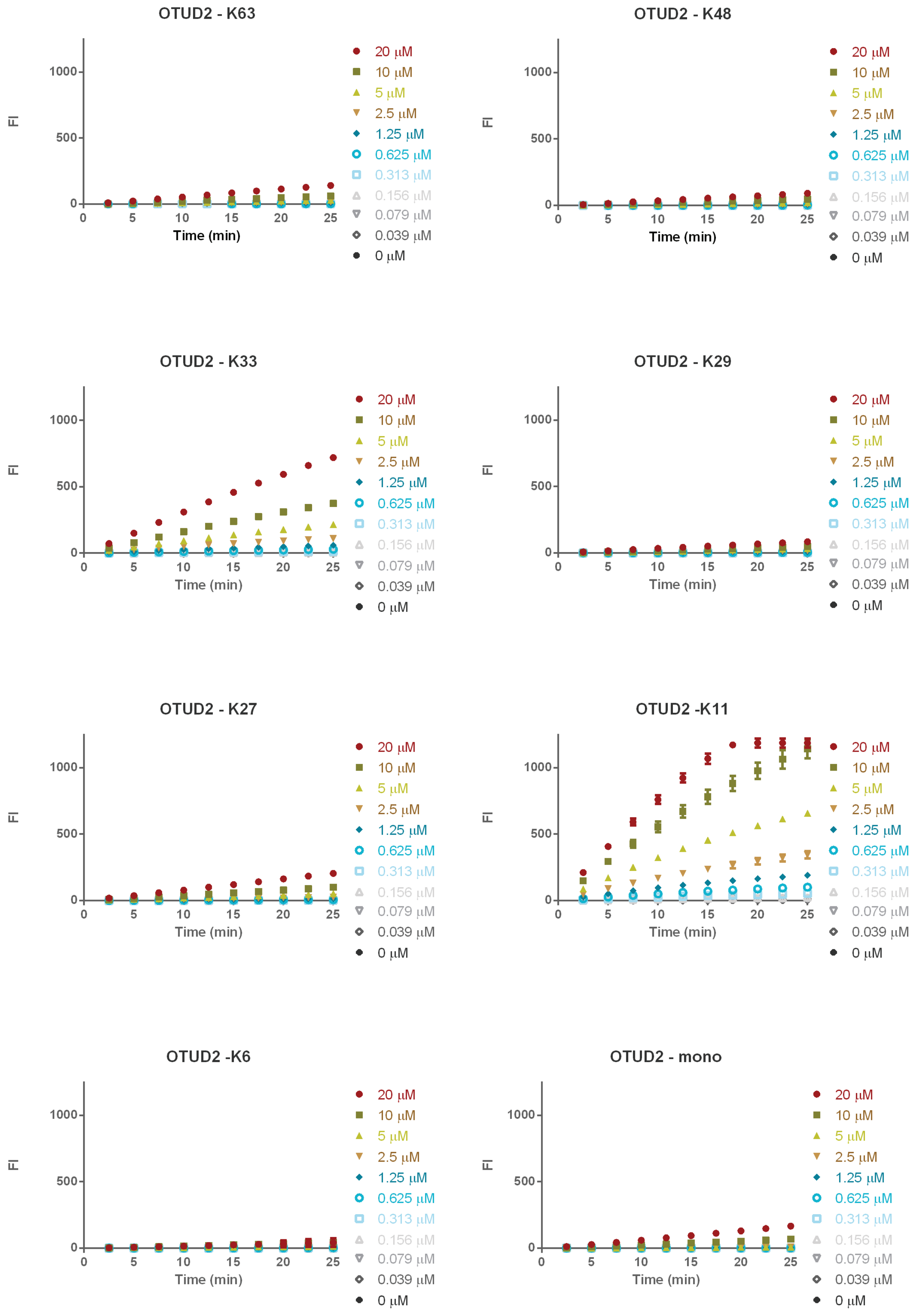

OTUD2 - K63
OTUD2 - K48
OTUD2 - K33
OTUD2 - K29
OTUD2 - K27
OTUD2 -K11
OTUD2 -K6
OTUD2 - mono
FI
1000
500
0
0
5
10
15
20
25
Time (min)
20 µM
10 µM
5 µM
2.5 µM
1.25 µM
0.625 µM
0.313 µM
0.156 µM
0.079 µM
0.039 µM
0 µM

Figure S4

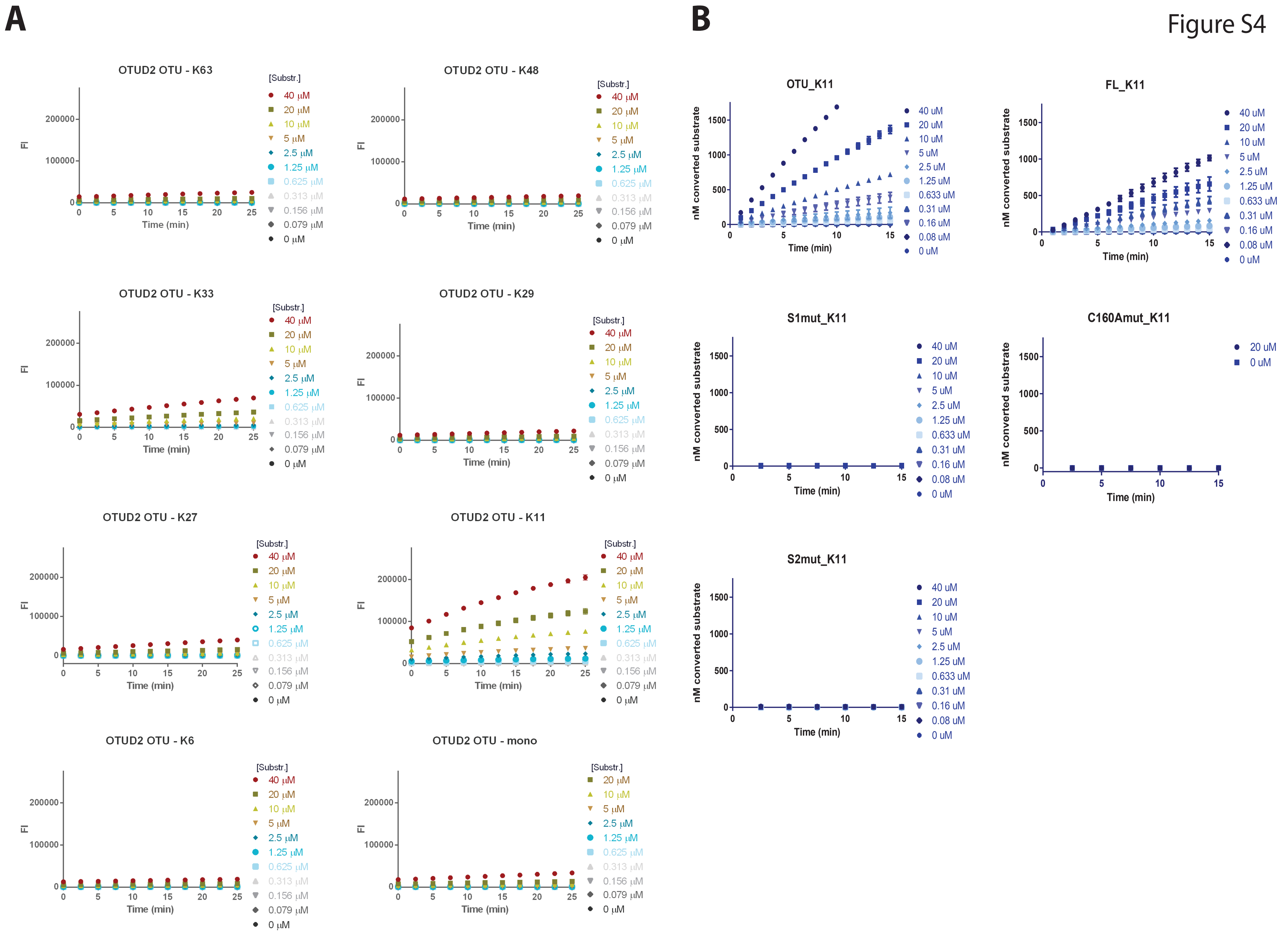
A
OTUD2 OTU - K63
OTUD2 OTU - K48
OTUD2 OTU - K33
OTUD2 OTU - K29
OTUD2 OTU - K27
OTUD2 OTU - K11
OTUD2 OTU - K6
OTUD2 OTU - mono
FI
Time (min)
[Substr.]
40 µM
20 µM
10 µM
5 µM
2.5 µM
1.25 µM
0.625 µM
0.313 µM
0.156 µM
0.079 µM
0 µM
B
OTU_K11
FL_K11
S1mut_K11
C160Amut_K11
S2mut_K11
nM converted substrate
Time (min)
40 uM
20 uM
10 uM
5 uM
2.5 uM
1.25 uM
0.633 uM
0.31 uM
0.16 uM
0.08 uM
0 uM

Figure S5

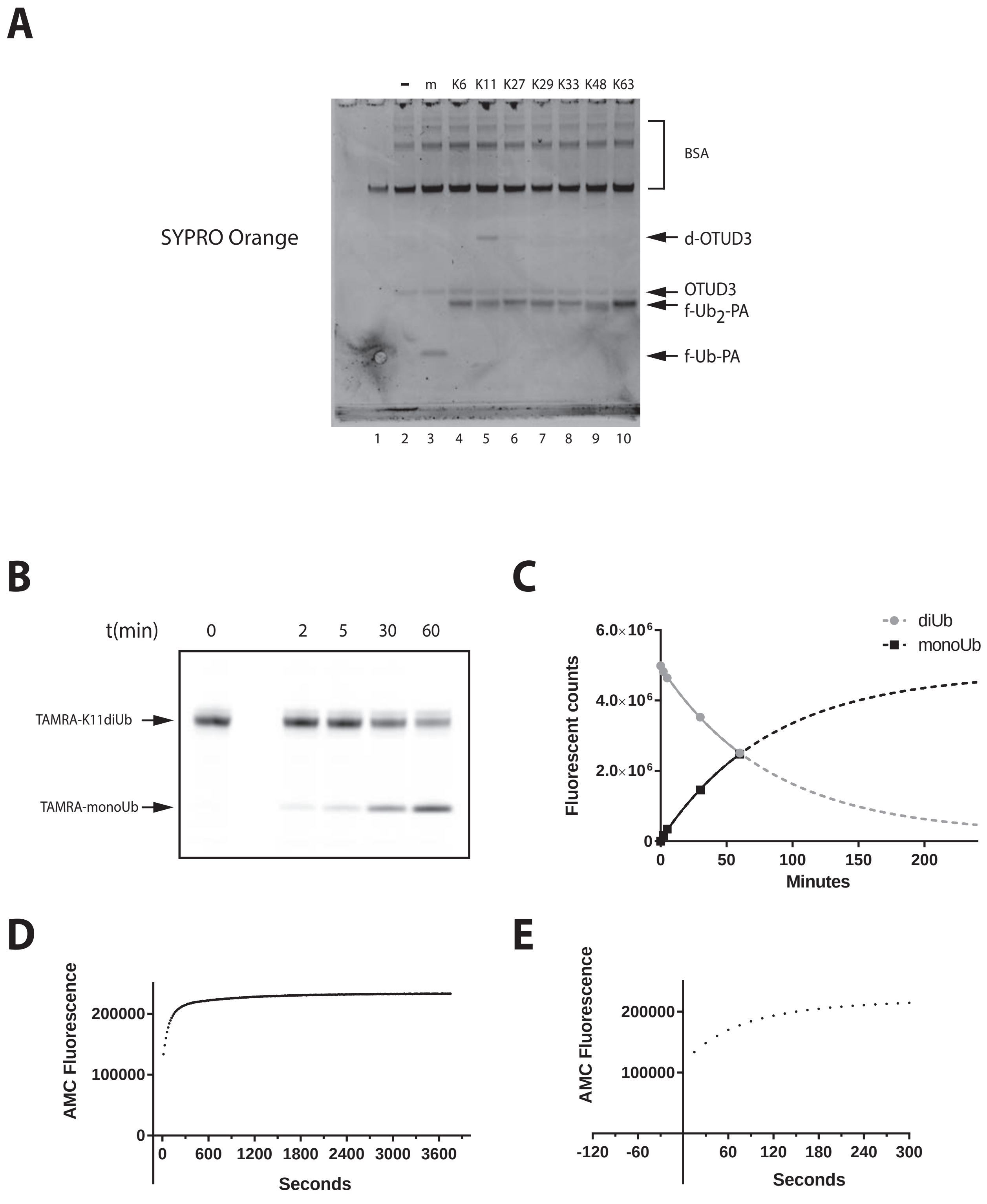

A
SYPRO Orange
–
m
K6
K11
K27
K29
K33
K48
K63
BSA
d-OTUD3
OTUD3
f-Ub2-PA
f-Ub-PA
1 2 3 4 5 6 7 8 9 10
B
t(min)
0
2
5
30
60
TAMRA-K11diUb
TAMRA-monoUb
C
diUb
monoUb
Fluorescent counts
6.0×10⁶
4.0×10⁶
2.0×10⁶
0
0 50 100 150 200
Minutes
D
AMC Fluorescence
200000
100000
0
0 600 1200 1800 2400 3000 3600
Seconds
E
AMC Fluorescence
200000
100000
-120 -60 60 120 180 240 300
Seconds

Figure S6

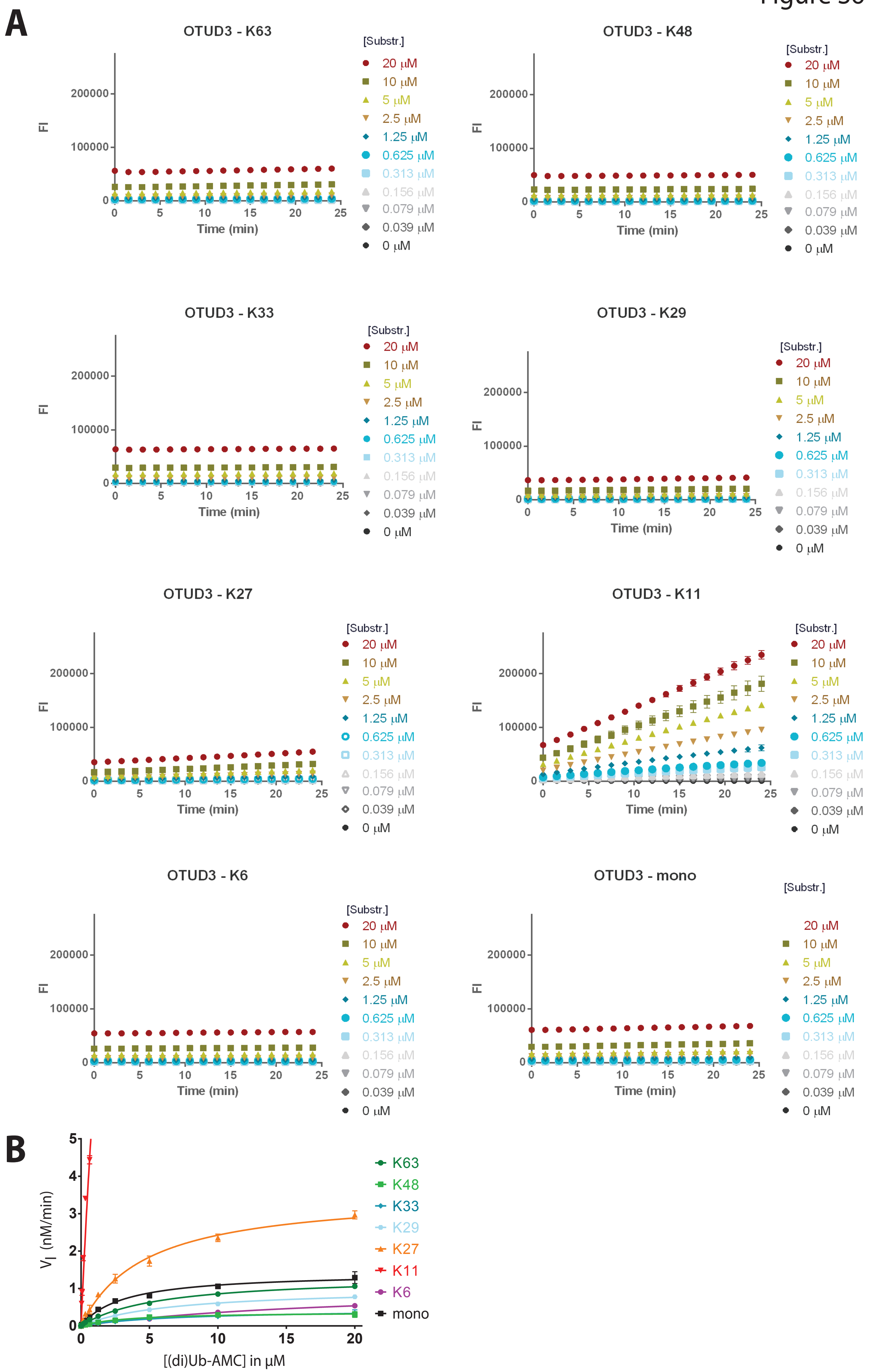
A
OTUD3 - K63
OTUD3 - K48
OTUD3 - K33
OTUD3 - K29
OTUD3 - K27
OTUD3 - K11
OTUD3 - K6
OTUD3 - mono
FI
Time (min)
[Substr.]
20 μM
10 μM
5 μM
2.5 μM
1.25 μM
0.625 μM
0.313 μM
0.156 μM
0.079 μM
0.039 μM
0 μM
B
V_I (nM/min)
[(di)Ub-AMC] in μM
K63
K48
K33
K29
K27
K11
K6
mono

Scheme S1

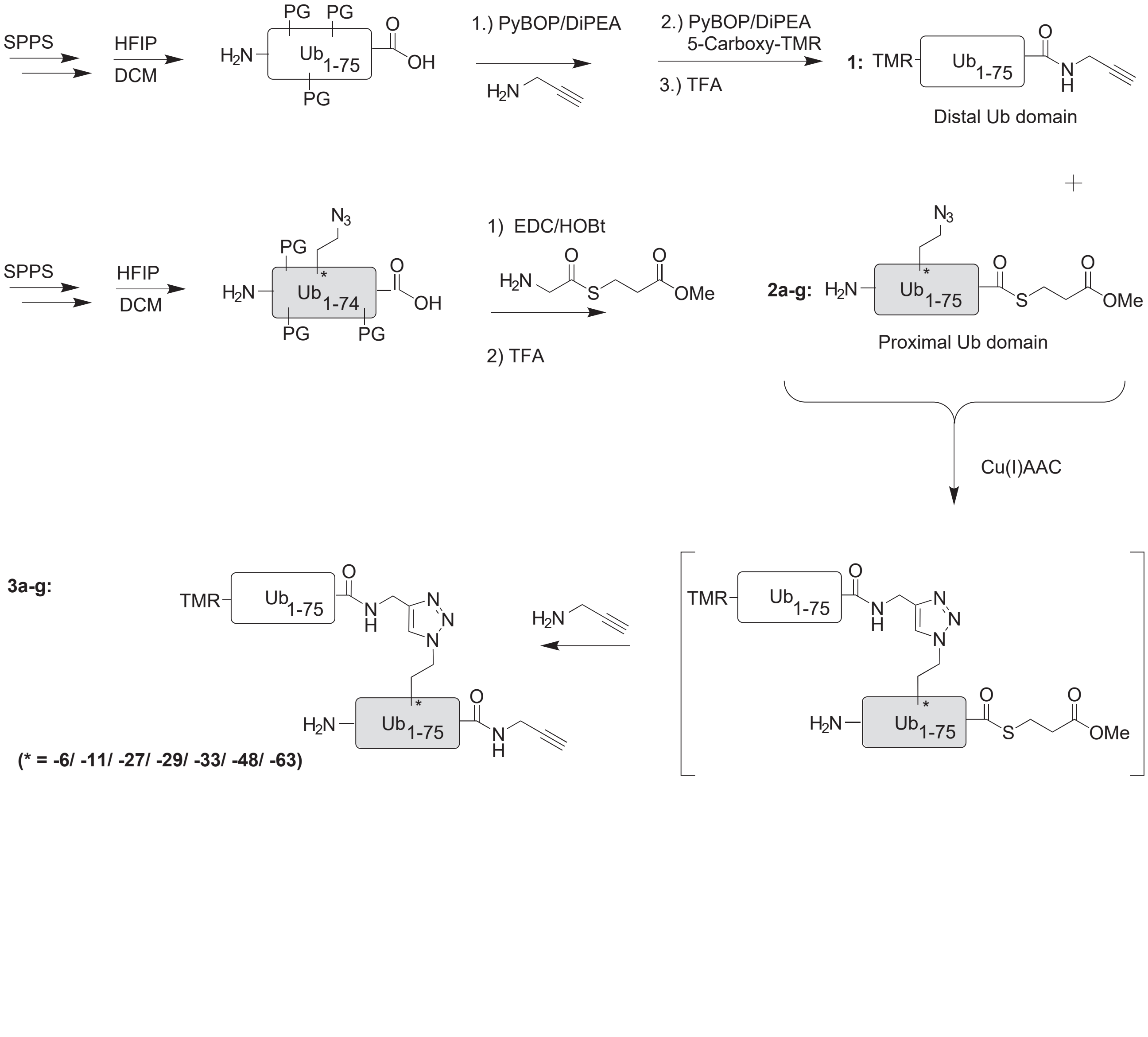

SPPS
HFIP
DCM
PG
PG
PG
H2N
Ub1-75
O
OH
1.) PyBOP/DiPEA
H2N
2.) PyBOP/DiPEA
5-Carboxy-TMR
3.) TFA
1:
TMR
Ub1-75
O
N
H
Distal Ub domain
+
N3
PG
PG
PG
*
Ub1-74
1) EDC/HOBt
S
OMe
2) TFA
2a-g:
Ub1-75
Proximal Ub domain
Cu(I)AAC
3a-g:
N
N
N
(* = -6/ -11/ -27/ -29/ -33/ -48/ -63)

Scheme S2

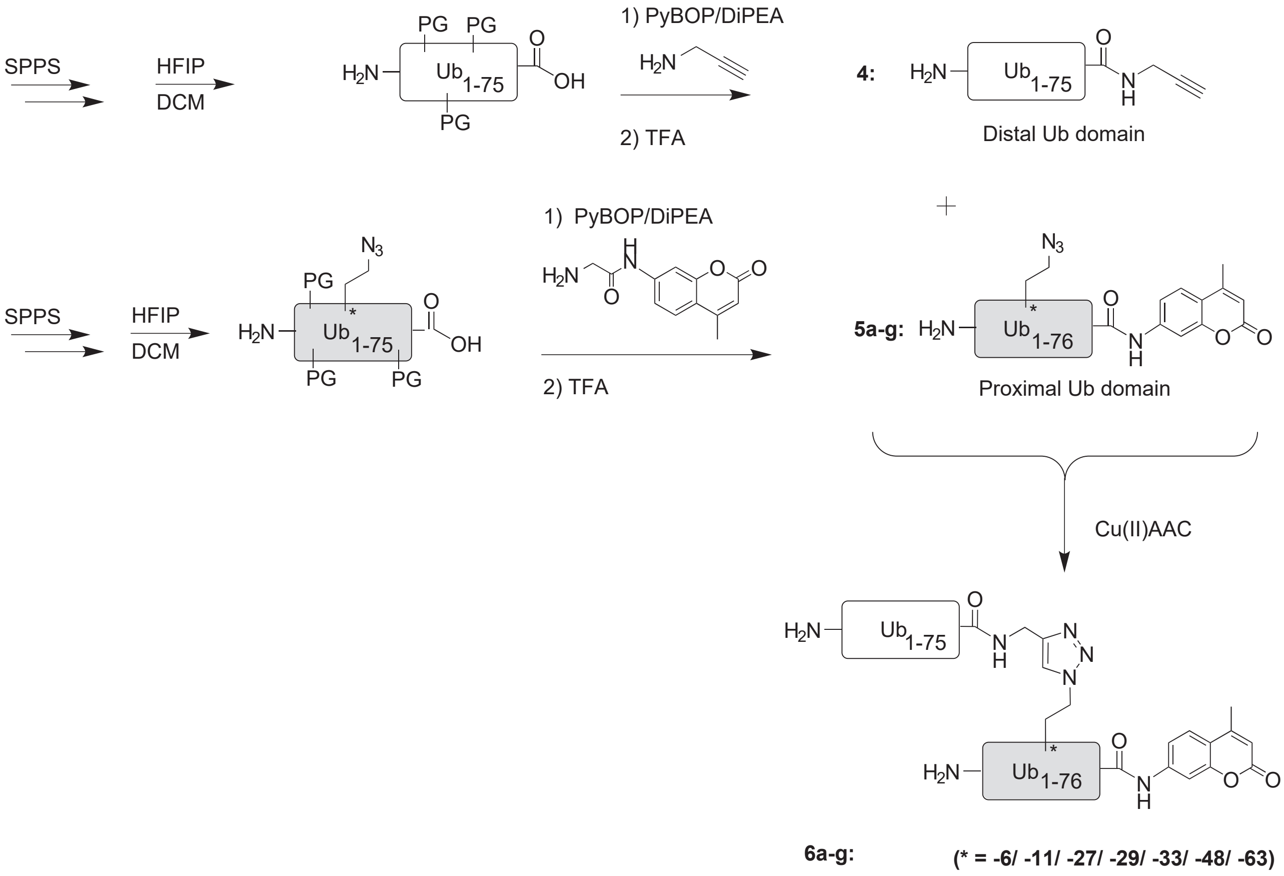
SPPS
HFIP
DCM
PG
H2N
Ub1-75
OH
1) PyBOP/DiPEA
H2N
2) TFA
4:
Distal Ub domain
N3
1) PyBOP/DiPEA
5a-g:
Ub1-76
Proximal Ub domain
Cu(II)AAC
6a-g:
(* = -6/ -11/ -27/ -29/ -33/ -48/ -63)

Supplemental Figure Legends

Figure S1. Full gels of Figure 3B showing that TAMRA-diUb probes reveal linkage-specific reactivity in EL4 lysates. (A) TAMRA-labeled diUb-PA probes were incubated at 37 °C for 30 minutes with EL4 cell lysate at a concentration of 5 µM. At indicated time points, samples were taken and collected for SDS-PAGE gel analysis. Time points are indicated in seconds (s) or in minutes (m). Gels were scanned at Ex/Em of 550/590 nm. USP14 is indicated by an arrow. (B) EL4 lysates were incubated at 30 °C for 40 minutes with buffer (-), 3.4 µM TAMRA-monoUb (m) or indicated TAMRA-diUb probes. Depending on type of gel and running buffer, for some diUb probes bands appear to run at a different molecular size or a double band is observed (*). USP14 bound to monoUb-PA (m) or diUb-PA (d) is indicated. f-$Ub_{(2)}$-PA indicates the unbound TAMRA-labeled (di)Ub probe.

Figure S2. Full details of OTUD2 linkage-specific reactivity with protease-resistant probes related to Figures 4 and 5. (A) Full SYPRO Orange stained gels from Figure 4A. Indicated are the positions of the unmodified OTUD2 enzyme and the TAMRA-labeled diUb-OTUD2 adducts (d-OTUD2), which are seen for some linkages as a band running slightly below bovine serum albumin (BSA), which was present in the assay mix. Especially for the K11-linked diUb-PA probe (lanes 9-12), the disappearance of the OTUD2 band is observed, in accordance with it reacting with the diUb-PA probe. (B) Full SYPRO Orange stained gels of Figure 5D. 0.1 µM of full-length (FL) OTUD2 or isolated OTU domains of WT OTUD2 (aa 147–314), an S1 site mutant (aa 147–314, AI200-201DD), an S2 site mutant (aa 147–314, I292Q, V295Q), and the catalytically inactive C160A mutant constructs were incubated with TAMRA-labeled K11-linked diUb-PA probes for 60 min at 1 µM. At indicated time points, samples were taken for analysis. OTUD2 or OTUD2 OTU, coupled to TAMRA-labeled diUb-PA (d) is indicated. Samples were analyzed by SDS-PAGE and fluorescence scanning at Ex/Em of 480/590 nm. f-Ub_2-PA indicates the unbound diUb probe fluorescently labeled with TAMRA. (C) Bands of full-length OTUD2 (FL) or OTUD2 OTU modified with TAMRA-labeled K11-linked diUb-PA probe in Figure 5D were quantified using Studio Image Lite and the data was fitted using one phase association curve. Quantification is displayed as arbitrary fluorescence units (AFU).

Figure S3. DiUb-AMC assay for full-length OTUD2 related to figures 4D and 4E. 15 nM OTUD2 was incubated for indicated time with Ub-AMC or the seven differently linked diUb-AMC substrates at different concentrations as indicated. The increase in fluorescence was measured at Ex/Em of 360/450 nm.

Figure S4. DiUb-AMC assays for the wild-type full length OTUD2 and isolated OTU domain of OTUD2 mutants, related to Figure 5C and 5E. (A) 15 nM OTUD2 was incubated for indicated time with Ub-AMC or the seven differently linked diUb-AMC substrates at different indicated concentrations. The increase in fluorescence was measured at Ex/Em of 360/450 nm. (B) 15 nM wild-type OTUD2 FL, wild-type OTUD2 OTU, or OTUD2 OTU mutants MutS1, MutS2 and C160A were incubated for indicated time with K11-linked diUb-AMC substrates at different indicated concentrations. The increase in fluorescence was measured at Ex/Em of 360/450 nm.

Figure S5 related to Figure 6. (A) 0.15 µM OTUD3 OTU was incubated with buffer (-), with TAMRA-labeled monoUb-PA (m) or the seven differently linked diUb-PA probes at 1 µM for 4 min. Gels were scanned at Ex/Em of 480/590 nm. Indicated are the OTUD3 OTU enzyme, which runs slightly above the diUb-PA probes, and the diUb-OTUD3 OTU adduct that is observed for the K11 linkage (d-OTUD3). Bovine serum albumin (BSA) was present in the assay mix. f-$Ub_{(2)}$-PA indicates the unbound TAMRA-labeled (di)Ub probe. (B) 7 µM TAMRA-labeled K11-linked diUb was incubated with 2 µM OTUD3 OTU. At indicated time points, samples were taken for gel analysis to monitor conversion of diUb to mUb. (C) Band from (B) were quantified and plotted to determine the half-time of the reaction, which is approximately 60 minutes. (D) 10 µM K11-linked diUb-AMC was incubated with 2 µM OTUD3 OTU and fluorescence as a result of cleavage of the AMC moiety was measured over time. Half-time was estimated to be between 90 and 120 seconds. (E) Plot showing first 300 seconds of (D).

Figure S6. DiUb-AMC assay for OTUD3 OTU related to Figure 6C and 6D. (A) 15 nM OTUD3 OTU was incubated for indicated time with Ub-AMC or the seven differently linked diUb-AMC substrates at different concentrations as indicated. The increase in fluorescence was measured at Ex/Em of 360/450 nm. (B) Michaelis-Menten plot shown in Figure 6D, but zoomed in to show the curves for monoUb-AMC and K27-linked diUb-AMC.

Scheme S1, related to Figure 2. Synthetic Scheme of TAMRA-diUb-PA probes **3**. Solid Phase Peptide Synthesis (SPPS) of Ub_{75} is followed by HFIP mediated release from the solid support, leaving all side-chain protecting groups (PG) in place. The C-terminal carboxylic acid is activated using PyBOP/DiPEA and propargylamine is coupled. Global deprotection is effected by trifluoroacetic acid (TFA) treatment resulting in TAMRA-Ub-PA **1**. Similarly, HFIP-mediated release from the solid support, liberates azido-ornithine Ub_{74}. C-terminal activation followed by coupling to methyl-3-(glycylthio)-propionate and concomitant TFA deprotection yields azido-ornithine thioesters **2**. Subsequent Cu(I)AAC of alkyne **1** and azide **2** results in the intermediate diUb-thioester, that is treated with propargylamine to yield final diUb-PA probes **3.**

Scheme S2, related to Figure 2. Synthetic Scheme of diUb-AMC probes **6**.
Solid Phase Peptide Synthesis (SPPS) of Ub_{75} is followed by HFIP mediated release from the solid support, leaving all side-chain protecting groups (PG) in place. The C-terminal carboxylic acid is activated using PyBOP/DiPEA and propargylamine is coupled. Global deprotection is effected by trifluoroacetic acid (TFA) treatment resulting in Ub-PA **4**. Similarly, HFIP-mediated release from the solid support, liberates azido-ornithine Ub_{75}. C-terminal activation followed by coupling to glycyl-7-amido-4-methylcoumarine and concomitant TFA deprotection yields azido-ornithine Ub-AMC mutants **5.** Subsequent Cu(II)AAC of alkyne **4** and azide **5** yields final diUb-AMC substrates **6.**

Supplemental Experimental Procedures

General experimental procedures

Chemical reagents were obtained from Sigma-Aldrich, Fluka and Acros of the highest available grade and used without further purification. Peptide synthesis reagents were purchased from Novabiochem. LC-MS measurements were performed on a Waters 2795 Separation Module (Alliance HT), Waters 2996 Photodiode Array Detector (190-750nm), Phenomenex Kinetex C18 (2.1x50, 2.6 µm) and LCT™ Orthogonal Acceleration Time of Flight Mass Spectrometer (Micromass). Samples were run using 2 mobile phases: A = 1% acetonitrile, 0.1% formic acid in water and B = 1% water and 0.1% formic acid in acetonitrile. Data processing was performed using Waters MassLynx Mass Spectrometry Software 4.1 (deconvolution with MaxEnt I function).

General Purification Procedure of Ub-mutants

The crude Ub derivatives were dissolved in a minimal amount of warm DMSO and then diluted by addition to Milli-Q water. A stock solution of NaOAc buffer was added to a final concentration of 50 mM NaOAc pH 4.5 (the final DMSO concentration is kept between 2 – 10%). Next, the peptide was purified by cation exchange chromatography using Workbeads 40S (Cation Exchange) material with a 0 → 1 M NaCl gradient in 50 mM NaOAc pH 4.5. Pure (>90%) fractions, as indicated by LC-MS, were pooled and further purified by RP-HPLC using a Waters XBridge OBD (150x30) C18 column with a linear gradient between 20-45% B over 25 minutes. (A = 95/5/0.05 H_2O/Acetonitrile/Trifluoroacetic acid; B = 5/95/0.05 H_2O/Acetonitrile/ Trifluoroacetic acid). Pure fractions (>95%), as judged by LC-MS were pooled, lyophilized and used as such.

Synthesis of mono-Ubiquitin precursors

Synthesis of TMR-Ub$_{1\text{-}75}$-PA ***1*** *(See Scheme S1)*

Ubiquitin$_{1\text{-}75}$ was synthesized using a solid phase peptide synthesis protocol as was described previously (El Oualid et al., 2010), using preloaded trityl resin; TentaGel® R TRT-Gly Fmoc (Rapp Polymere GmbH; RA1213). After SPPS synthesis an aliquot of resin was treated with TFA cleavage cocktail (90% TFA, 5% H_2O, 2.5% triisopropylsilane, 2,5% phenol) and product was precipitated in cold diethylether:pentane (3:1). The identity was confirmed by LC-MS analysis. The *N*-terminal Fmoc-group was then removed from the resin bound polypeptide by treatment with 20% piperidine in N-Methyl-2-pyrrolidone (NMP) (3 x 10 mL, 10 minutes). The protected peptide was then released from the resin by incubation for 30 minutes with hexafluoroisopropanol:DCM mixture (1:4, v/v) to afford a globally protected Ub$_{1\text{-}75}$ polypeptide. After evaporation of the solvent the solid was coevaporated with dichloroethane (DCE) three times to remove traces of hexafluoroisopropanol (HFIP) which may lead to undesired HFIP ester formation in the following step. The peptide (25 µmol) was dissolved in DCM (1 mL/ 5 µmol), and 5 eq. PyBOP, 5 eq. triethylamine and 10 eq. propargylamine were reacted for 16 hours. The reaction mixture was concentrated and excess propargylamine was removed by co-evaporation with DCM and toluene followed by dissolution of the protected Ub-PA in DCM and extraction with 1M $KHSO_4$ (2 times) and sat. aq. NaCl before drying the organic layer using sodium sulfate and concentration. The resulting off-white solid was further dried overnight under high vacuum. Subsequently, 5 eq. of 5-Carboxy-Tetramethylrhodamine (TMR) was pre-activated in anhydrous DMF by the addition of 5 eq. PyBOP and 5 eq. triethylamine for 5 minutes prior to the addition of protected Ub-propargyl to the reaction mixture. After reaction for 16 hours, the reaction mixture was concentrated and the deep purple TMR-Ub$_{1\text{-}75}$-PA was deprotected using TFA cleavage mixture for 2.5 hours. The crude polypeptide was collected after precipitation from cold diethyl ether/pentane (3:1), centrifugation (1000 *g*, 5 minutes) and washing 3 times with cold diethyl ether and further purified as described in the 'G*eneral Purification Procedure*' above.

Synthesis of (Azido-ornithine)Ub-thioester ***2a-g*** *(See Scheme S1)*

Ubiquitin$_{1\text{-}74}$ was synthesized using a solid phase peptide synthesis protocol as was described previously (El Oualid et al., 2010) incorporating Fmoc-L-azido-ornithine as substitute on any of the desired lysine positions. After solid phase synthesis and release from the resin using HFIP/DCM (1:4, v/v) the solid (25 µmol) was dissolved in 6 mL of chloroform and cooled to -10 °C while stirring. Trifluoroethanol (2 mL) was added which resulted in a milky solution. These conditions were selected in order to prevent epimerization of Arg74 during the subsequent coupling protocol as reported by Sakakibara (Sakakibara, 1995). To this suspension 3 eq. of methyl-3-(glycylthio)proanoate, 3 eq. EDC and 3 eq. HOBt were added. (To avoid the reduction of the azide by free thiols we decided to introduce the thioester as the preformed glycylthioester). This was stirred for 10 minutes before removing the icebath. The reaction was

completed after 3 hours as indicated by a test cleavage (as described above). Solvents were removed under reduced pressure and the peptide was deprotected using TFA cleavage mixture for 2.5 hours. The crude was collected after precipitation from cold diethyl ether/pentane (3:1), centrifugation (1000 *g*, 5 minutes) and washing 3 times with cold diethyl ether and further purified as described in the 'G*eneral Purification Procedure*' above.

*Synthesis of Ub-PA **4** (See Scheme S2)*

$Ub_{1\text{-}75}$-PA was prepared as described previously (Ekkebus et al., 2013). Briefly, after SPPS and release from the resin using HFIP/DCM (1:4, v/v) the protected polypeptide $Ub_{1\text{-}75}$ (25 µmol) was dissolved in DCM (1 mL/ 5 µmol), and 5 eq. PyBOP, 5 eq. triethylamine and 10 eq. propargylamine were reacted for 16 hours. The reaction mixture was concentrated and deprotected using TFA cleavage mixture for 2.5 hours. The crude polypeptide was collected after precipitation from cold diethyl ether/pentane (3:1), centrifugation (1000 *g*, 5 minutes) and washing 3 times with cold diethyl ether and further purified as described in the 'G*eneral Purification Procedure*' above.

*Synthesis of (Azido-ornithine)Ub-AMC **5a-g** (See Scheme S2)*

After SPPS and release from the resin using HFIP/DCM (1:4, v/v) the protected polypeptide $Ub_{1\text{-}75}$ (5 µmol) was dissolved in DCM (1 mL/ 5 µmol), and 5 eq. PyBOP, 5 eq. triethylamine and 10 eq. glycinyl-AMC were reacted for 16 hours. The reaction mixture was concentrated and deprotected using TFA cleavage mixture for 2.5 hours. The crude polypeptide was collected after precipitation from cold diethyl ether/pentane (3:1), centrifugation (1000 x *g*, 5 minutes) and washing 3 times with cold diethyl ether and further purified as described in the 'G*eneral Purification Procedure*' above.

Synthesis of diUb probes

*Synthesis of diubiquitin PA probes **3a-g** using Cu(I)AAC-reaction (See Scheme S1)*

TMR-Ub-PA **1** and Ub-thioester mutants **2a-g** were dissolved in warm DMSO at a concentration of 50 mg/mL (a microBCA kit was used to determine relative protein concentrations). The CuAAC reactions were performed under denaturing conditions in 8M Urea, 100 mM phosphate buffer pH 7.

It is critical to use high quality Cu(I)Br in these experiments, by stirring 99% CuBr in glacial acetic acid overnight at 1:10 v/v powder:solvent. The suspension was filtered resulting in a greenish filtrate and an off-white residue that was subsequently washed with ethanol and dried under reduced pressure at 50 °C followed by high vacuum for 16 hours. The Cu(I)Br was stored under inert (N_2) atmosphere.

In a typical reaction 100 µL of Ub-thioester mutant (5 mg) was added to 1 mL of reaction buffer (8M Urea pH 7), followed by addition of 0.9 eq. TMR-Ub-PA. To the resulting solution 10 µL of catalyst solution containing 20 mg/mL Cu(I)Br in MeCN and 50 mg/mL TBTA-analogue (Zhou and Fahrni, 2004) in MeCN (2:3, v/v) was added followed by a short vortex, repeated in 5 minute intervals 5 times in total. After reactions were finished, as judged by LC-MS (~ 1 hour), the reaction was quenched by the addition of 100 µL of 0.5 M EDTA, pH 7.0. A PD-10 desalting column (GE Lifesciences) was equilibrated with reaction buffer according to manufacturer's protocol and the sample buffer exchanged to remove the catalyst.

The volume of the resulting solution was adjusted to 3 mL by the addition of extra reaction buffer and 168 µL 2M HCl was added to prevent overshoot of pH during propargylamine addition. 42 µL propargylamine was added to initiate the thioester-to-propargylamine exchange. The reaction was allowed to proceed overnight at 4 °C and product was purified using RP-HPLC; Waters Atlantis T3 C18 30x250 5µm running at 18 mL/min. Solvent A is water with 0.05% TFA, Solvent B is Acetonitrile with 0.05% TFA. Gradient in **time** → *%B*: **0** → *10%,* **1** → *10%,* **2.50** → *20%,* **7.50** → *32%,* **27.50** → *42%,* **28** → *95%*. Fractions containing product were identified using LC-MS analysis, pooled and lyophilized. The semi-pure product, containing some mono-Ub remnants was dissolved in warm DMSO (50 µL) and diluted into Milli-Q (850 µL). After careful mixing a 10x gel filtration buffer stock solution was added to reach a final concentration of 150 mM NaCl, 50 mM TRIS.HCl pH 7.4. Samples were purified over a Superdex 75 pg 16/600 column (GE), which affords baseline separation of mono and diubiquitin species, as well as some higher molecular weight aggregates. All seven TAMRA-diUb-PA probes **3** were verified by analytical LCMS.

*Synthesis of diubiquitin-AMC probes **6a-g** using Cu(II)AAC-reaction (See Scheme S2)*

Ub-PA **4** and (Azido-ornithine)Ub-AMC mutants **5a-g** were dissolved in warm DMSO at a concentration of 5 mg/mL for each Ub respectively. The CuAAC reactions were performed under denaturing conditions in 8M Urea, 100 mM phosphate buffer pH 7.
In a typical reaction 75 µL of Ub-containg DMSO stock was added to 1275 µL of reaction buffer (8M Urea, pH 7), followed by addition of 150 µL catalyst solution containing 25 mg/mL Cu(II)SO_4 in MQ, 120 mg/mL Sodium Ascorbate in MQ and 52 mg/mL TBTA-analogue (Zhou and Fahrni, 2004) in MeCN (1:1:1, v/ (Zhou, 2004) in MeCN (1:1:1, v/v/v) followed by a short vortex, repeated 2 times in total after 15 min intervals. After reactions were finished, as judged by LC-MS (ranging from 30 min to 2 hour, dependent on the azido-ornithine position), the reaction was quenched by the addition of 100 µL of 0.5 M EDTA, pH 7.0. After buffer exchange to 150mM NaCl, 50 mM TRIS.HCl pH 7.6, samples were purified over a Superdex 75 pg 16/600 column (GE). Analytical LCMS data was obtained for all probes. All seven diUb-AMC substrates **6** were verified by analytical LCMS.

Synthesis of TAMRA-labeled native K11-linked diUb

TAMRA labeled diUb^{K11} was generated using a procedure based on previously reported protocols (El Oualid et al., 2010). Briefly, synthetic N-terminal TAMRA labeled Ub thioester (25 mg/mL) and synthetic K11-thiolysine Ub (25 mg/mL) were ligated in 8 M Gdn.HCl/ 200 mM NaH_2PO_4, pH 7.6 in the presence of 1.2 M mercaptophenyl acetic acid (MPAA) for 16 hours at 37 ºC. After HPLC and size exclusion chromatography (SEC), the resulting TMR-Ub_2^{K11} was desulfurized in 8 M Gdn.HCl/200 mM NaH_2PO_4, pH 6.8 in the presence of 200 mM TCEP, 75 mM GSH (reduced form) and 75 mM of the radical initiator VA-044. Concomitant HPLC and subsequent SEC-purification resulted in the target compound: TAMRA-Ub_2^{K11}. The TAMRA-K11-linked diUb substrate was verified by analytical LCMS.

Concentration determination of TMR-labeled probes

5-TAMRA absorption was measured at 553 nm using Nanodrop. Concentrations were calculated based on weighed 5-TAMRA-linked Lysine-Glycine. Additionally, labeled mono and diUb probes were adjusted for fluorescent signal of di- or monoUb bands by gel analysis using the ProXpress imaging system.

Supplemental references

Sakakibara, S. (1995). Synthesis of large peptides in solution. Biopolymers *37*, 17-28.
Zhou, Z., and Fahrni, C.J. (2004). A fluorogenic probe for the copper(I)-catalyzed azide-alkyne ligation reaction: modulation of the fluorescence emission via 3(n,pi)-1(pi,pi) inversion. Journal of the American Chemical Society *126*, 8862-8863.
