# Supplementary material for: Non-hydrolyzable Diubiquitin Probes Reveal Linkage-Specific Reactivity of Deubiquitylating Enzymes Mediated by S2 Pockets
Source: Cell Chem Biol. 2016 Apr 21;23(4):472–82. doi: 10.1016/j.chembiol.2016.03.009 (PMC4850247; doi:10.1016/j.chembiol.2016.03.009)

Analysis S1

A

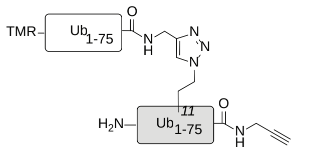

TAMRA-labeled K11-linked diUb-propargylamide probe

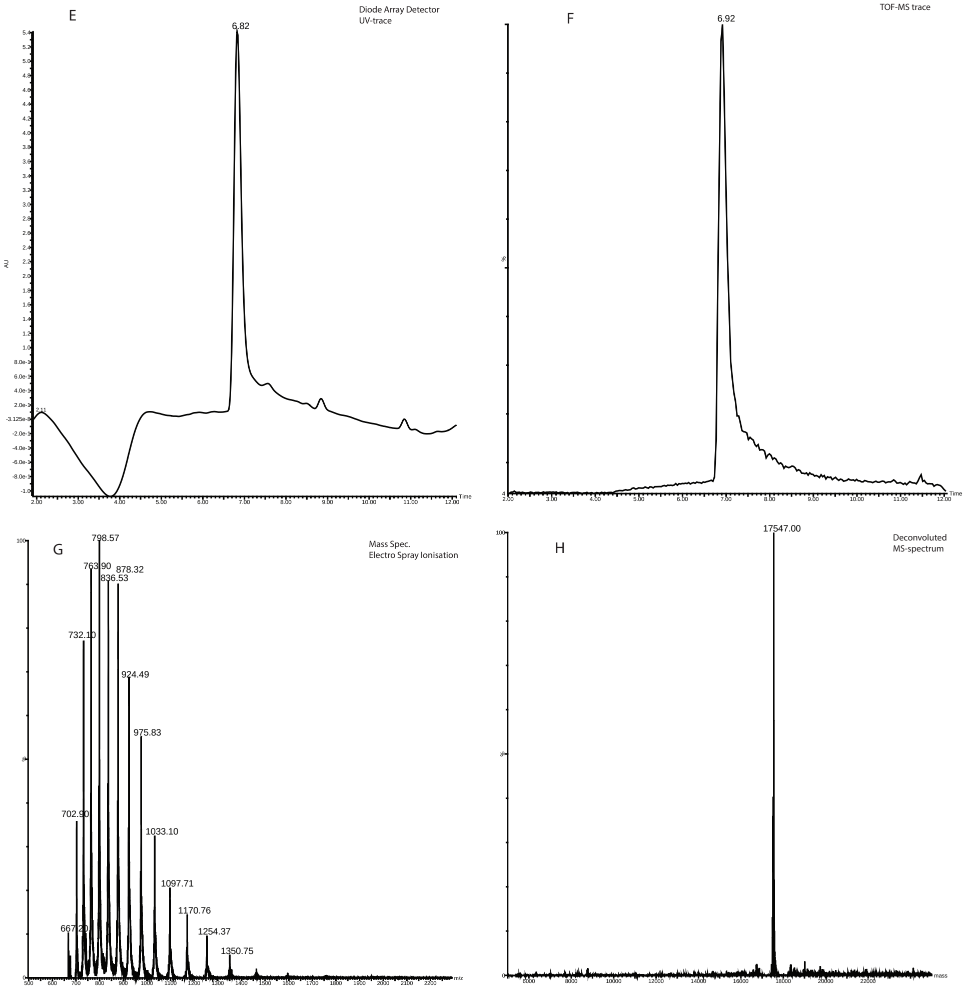

B

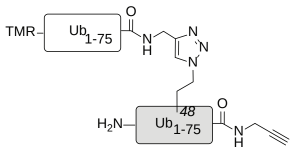

TAMRA-labeled K48-linked diUb-propargylamide probe

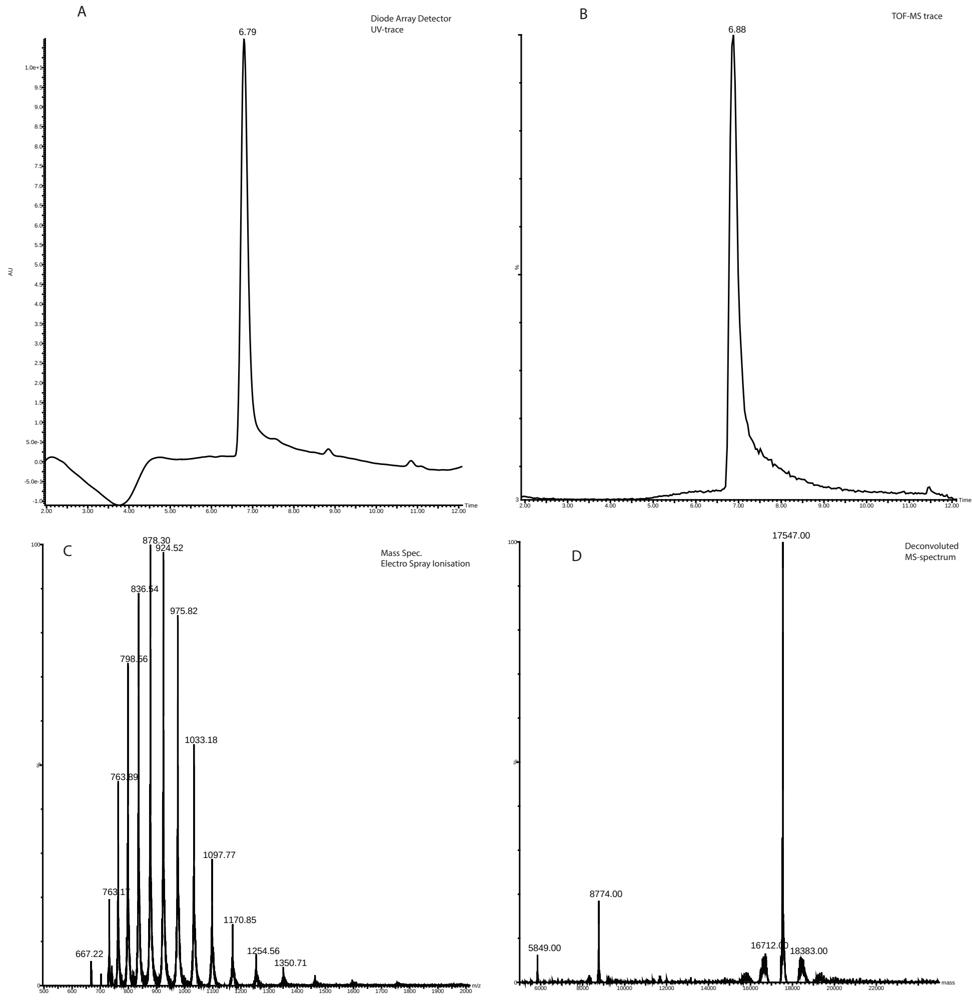

Supplement: Data S1. Analytical Data of diUb-PA Probes and diUb-AMC Substrates [file mmc2.zip › Analysis-S1a.pdf]
