# Supplementary figures and images for: Non-hydrolyzable Diubiquitin Probes Reveal Linkage-Specific Reactivity of Deubiquitylating Enzymes Mediated by S2 Pockets
Source: Cell Chem Biol. 2016 Apr 21;23(4):472–82. doi: 10.1016/j.chembiol.2016.03.009 (PMC4850247; doi:10.1016/j.chembiol.2016.03.009)

Analysis S2

A

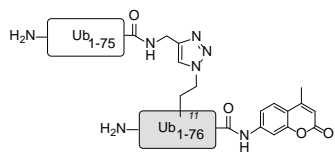

K11-linked diUb-AMC substrate

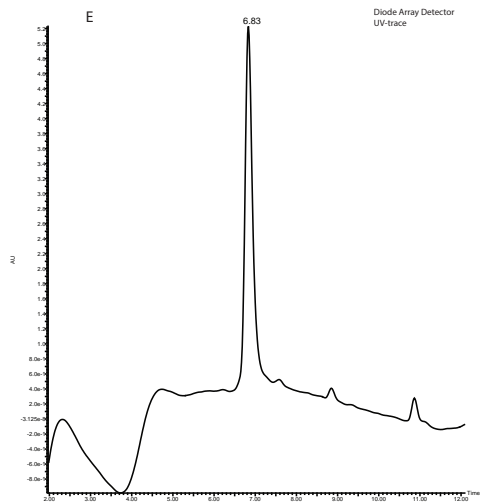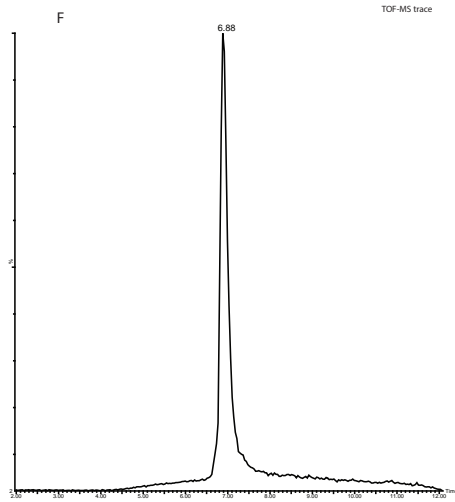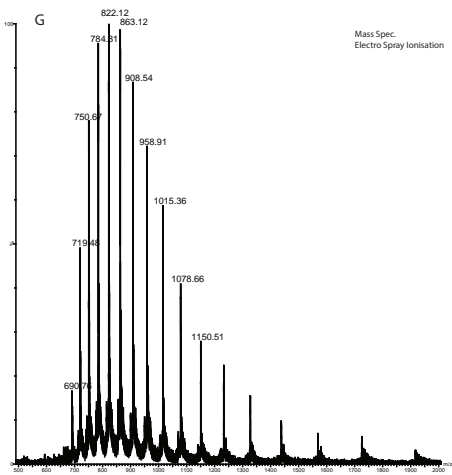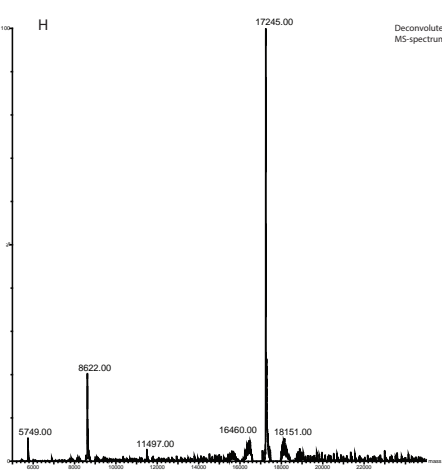

B

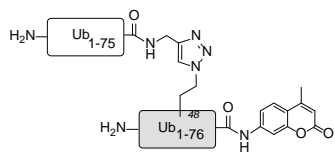

K48-linked diUb-AMC substrate

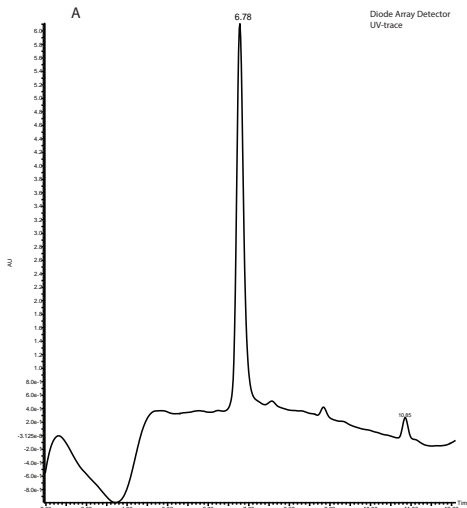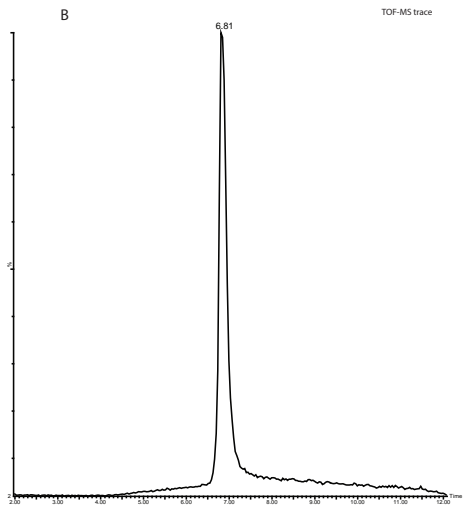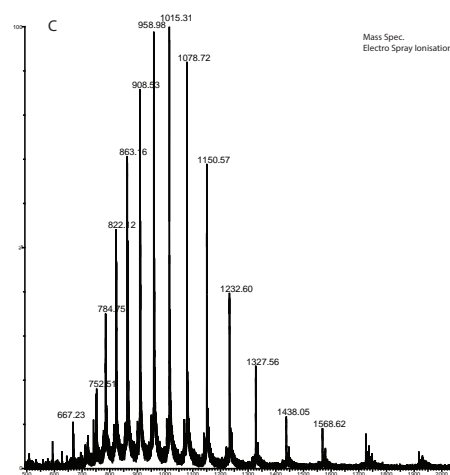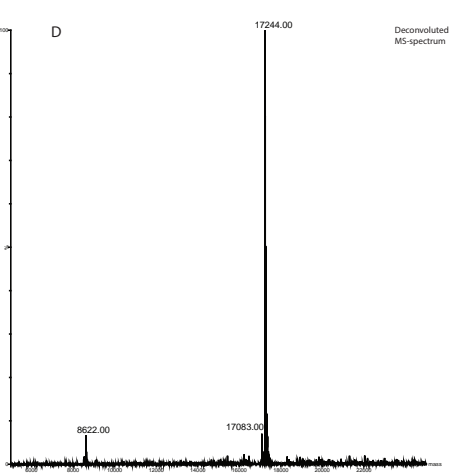

Supplement: Data S1. Analytical Data of diUb-PA Probes and diUb-AMC Substrates [file mmc2.zip › Analysis-S2a.pdf]

# Analysis S3

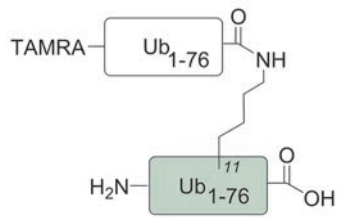

TAMRA-labeled native K11-linked diUb

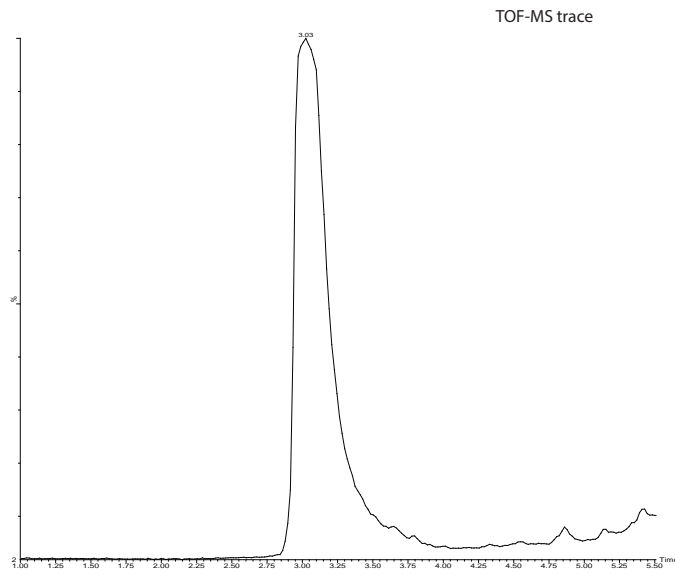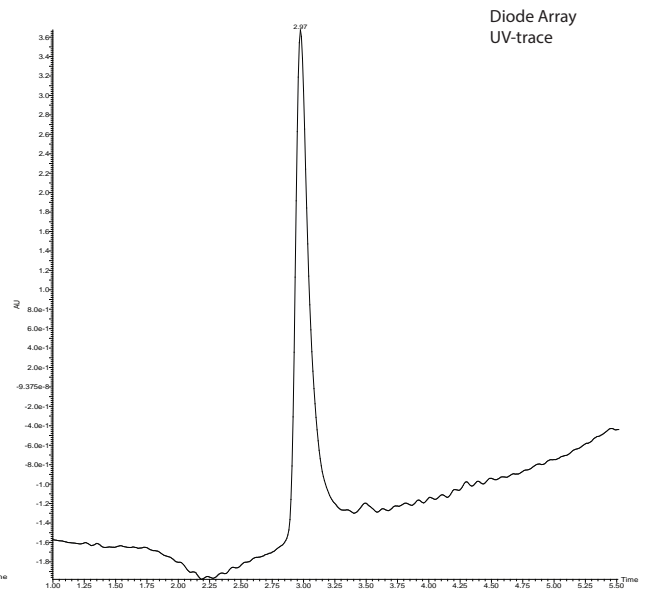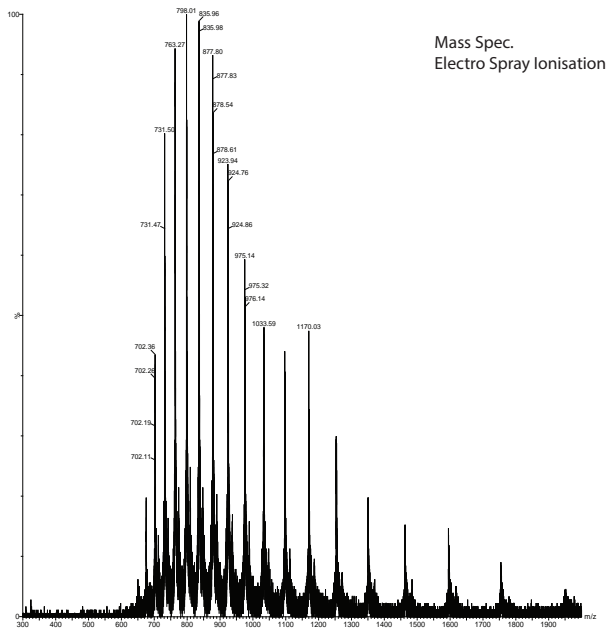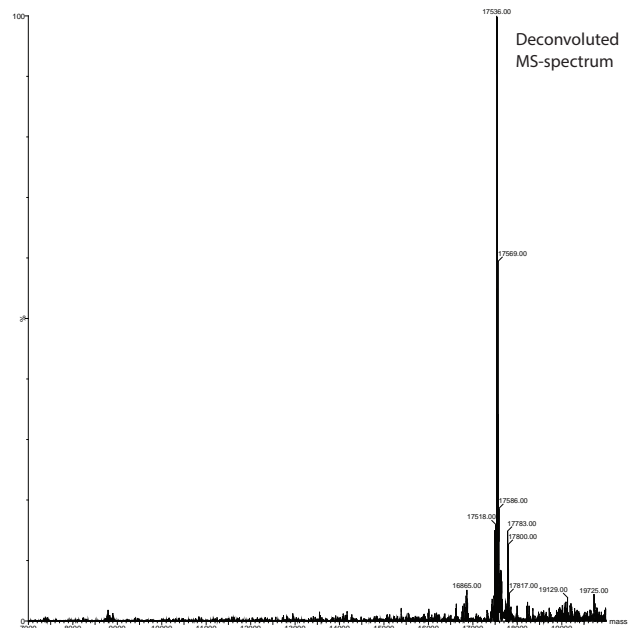

Supplement: Data S1. Analytical Data of diUb-PA Probes and diUb-AMC Substrates [file mmc2.zip › Analysis-S3a.pdf]
